# Surgical video workflow analysis via visual-language learning

**DOI:** 10.1038/s44401-024-00010-3

**Published:** 2025-01-25

**Authors:** Pengpeng Li, Xiangbo Shu, Chun-Mei Feng, Yifei Feng, Wangmeng Zuo, Jinhui Tang

**Affiliations:** 1 School of Computer Science and Engineering, Nanjing University of Science and Technology, Nanjing, China; 2 Institute of High Performance Computing (IHPC), Agency for Science, Technology and Research (A*STAR), Singapore, Republic of Singapore; 3 The First School of Clinical Medicine, Nanjing Medical University, Nanjing, China; 4 Department of General Surgery, The First Affiliated Hospital of Nanjing Medical University, Nanjing, China; 5 Faculty of Computing, Harbin Institute of Technology, Harbin, China

**Keywords:** Machine learning, Computer science

## Abstract

Surgical video workflow analysis has made intensive development in computer-assisted surgery by combining deep learning models, aiming to enhance surgical scene analysis and decision-making. However, previous research has primarily focused on coarse-grained analysis of surgical videos, e.g., phase recognition, instrument recognition, and triplet recognition that only considers relationships within surgical triplets. In order to provide a more comprehensive fine-grained analysis of surgical videos, this work focuses on accurately identifying triplets <*instrument*, *verb*, *target*> from surgical videos. Specifically, we propose a vision-language deep learning framework that incorporates intra- and inter- triplet modeling, termed I^2^TM, to explore the relationships among triplets and leverage the model understanding of the entire surgical process, thereby enhancing the accuracy and robustness of recognition. Besides, we also develop a new surgical triplet semantic enhancer (TSE) to establish semantic relationships, both intra- and inter-triplets, across visual and textual modalities. Extensive experimental results on surgical video benchmark datasets demonstrate that our approach can capture finer semantics, achieve effective surgical video understanding and analysis, with potential for widespread medical applications.

## Introduction

In medical surgery, particularly minimally invasive surgery, e.g., laparoscopic cholecystectomy, precise surgical decision-making is crucial. However, surgeons still face technical challenges due to the loss of direct visual and tactile perception^1,2^. These challenges have driven the development of intelligent technologies for understanding surgical video workflow. The existing research in surgical video workflow analysis mainly focuses on coarse-grained recognition. For example, surgical phase recognition^3–8^ (refers to a general understanding of the broader surgical phases, such as identifying phases like “preparation” or “cleaning coagulation”), instrument recognition^9–11^ and gesture recognition^12–14^ lack detailed insights into the interactions among surgical instruments, verbs, and targets, thereby limiting the comprehensive and accurate analysis of surgical video workflow. To achieve a detailed and holistic understanding of surgical video workflow, surgical video action triplet recognition is explored^15^, which recognizes surgical actions as triplets of <*instrument*, *verb*, *target*>, enabling intra-operative contextual awareness and supporting surgeons in their decision-making processes.

**Fig. 1:**
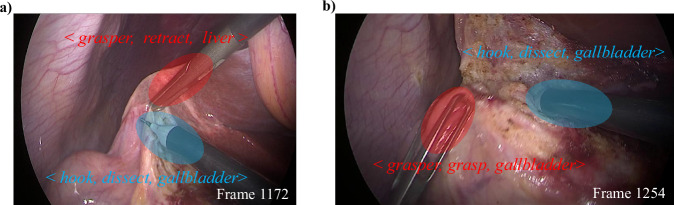
Illustration of multiple triplets within one frame in surgical video. As shown in (**a**, **b**), we demonstrate representative triplet co-occurrence in surgical videos.

A typical example of triplet recognition is shown in Fig. 1a. Two surgical triplets, i.e., <*grasper*, *retract*, *liver*> and <*hook*, *dissect*, *gallbladder*>, simultaneously appear in frame 1172 to represent the liver retraction with the grasper and the gallbladder dissection using the hook. Furthermore, it can be observed that in frame 1254 (Fig. 1b), although the triplet <*hook*, *dissect*, *gallbladder*> appears the same as in frame 1172, the co-occurring triplet is <*grasper*, *grasp*, *gallbladder*>. This situation demonstrates that understanding surgical scenes requires establishing intra-triplet relationships (e.g., in Fig. 1a: Frame 1172, <*hook*, *dissect*, *gallbladder*> represents the doctor using a hook to perform a dissection on the gallbladder. When recognizing it, we should be careful not to identify it as <*hook*, *dissect*, *liver*>), while also capturing the potential inter-triplet dynamic logical relationships (such as temporal relations, causal relations, etc.). Temporal relations involve the sequence and progression of surgical actions, helping to understand the progress of surgery. Causal relations describe how one surgical action influences or facilitates the occurrence of another, aiding in the establishment of the causal sequence and co-occurrence analysis in the surgical task.

Previous studies mainly employ compositional methods by individually modeling instruments, verbs, and targets^15–18^, such as Tripnet^15^, Attention Tripnet^17^, and RDV^17^. Moreover, some studies focus on the class imbalance characteristic in surgical triplets, Forest-GCN^19^ integrates GCN with a classification forest to better balance triplet classes in a long-tail distribution. Building on these approaches, Chen et al.^20^ introduce a triplet disentanglement (TD) framework for learning objective decomposition, while Xi et al. propose chain of thought Cot^21^ to illustrate an verb-centric surgical triplet recognition modeling scheme. However, the aforementioned methods lack sufficient modeling of the intra- and inter-relations among surgical video action triplets, and previous methods limit the modeling of task-specific semantics to a single modality, relying solely on textual information. Therefore, we aim to well learn the specific relational semantics (refers to understanding how different verbs, tools, and targets in surgery are related to each other, such as understanding how the “grasper” instrument relates to the “retracting liver” context) of triplet. To mitigate the limitation of single-modality modeling, our method combines a dual textual/visual graph to enrich relational sementics both intra- and inter-triplets, across visual and textual modalities. Recently, Vision-Language Models (VLMs) have obtained remarkable success in various vision tasks^22–24^. For the surgical video workflow analysis task, textual prompts have been utilized in the explicit reasoning process to guide the understanding of surgical videos, thereby improving recognition accuracy. However, directly utilizing traditional VLMs falls short in capturing the specific mutual intra- and inter-triplet relational semantics, thereby leading to erroneous matches and limiting the understanding of surgical videos.

**Fig. 2:**
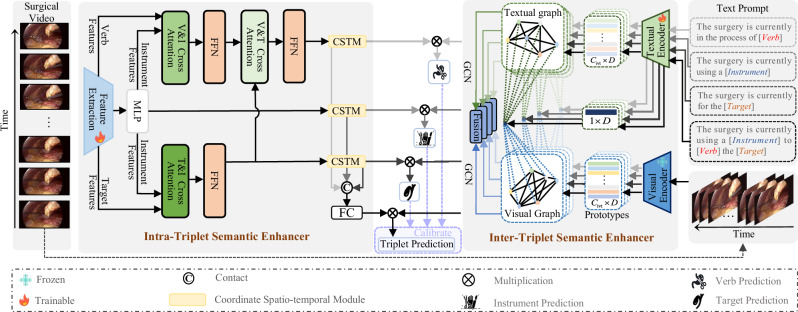
The overall architecture of the Vision-language deep learning model architecture, I^2^TM. (Left): Intra-Triplet Semantic Enhancer (Intra-TSE) enhances spatio-temporal visual relational semantics by utilizing intra-triplet interaction and the Coordinate Spatio-Temporal Module (CSTM). (Right): Inter-Triplet Semantic Enhancer (Inter-TSE) enhances inter-triplet relational semantics by jointly constructing textual and visual graphs. (Middle): I^2^TM aligns the visual-textual features of intra- and inter-triplet and calibrates the prediction of the triplet using the prediction of individual components.

In this study, to address the above problems, we introduce a new Triplet Semantic Enhancer (TSE) consisting of Intra-Triplet Semantic Enhancer (Intra-TSE) in the visual branch and an Inter-TSE in the textual branch, as shown in Fig. 2. In the visual branch, Intra-TSE employs cross-attention to facilitate interaction among all components of triplets based on the features extracted by the visual backbone. Subsequently, as shown in Supplementary Fig. 1, the Coordinate Spatio-Temporal Module (CSTM) is further utilized for capturing the spatio-temporal relational semantics in each component intra-triplet. In the textual branch, unlike previous methods^19,21^, Inter-TSE jointly constructs textual and visual graphs, as well as embeds the semantics of triplet textual prompts into both textual and visual spaces. Specifically, to establish relationships among triplets in textual space, we treat the textual features of specific categories as nodes in the textual graph and measure inter-class relationships by assessing the distance between their corresponding features as edges of the graph. Similarly, the visual graph aims to model relationships between different triplet relational semantics from the visual perspective, where nodes are constructed using the average features of the training samples from the same class. Subsequently, we integrate each textual prompt feature into the relational semantic of both textual and visual modalities using graph convolutional network (GCN)^25^, capturing the dynamic relational semantics among triplets, thereby enhancing the logical reasoning and understanding capabilities of surgical videos. To this end, we propose a framework by incorporating Intra- and Inter-Triplet Modeling to analyze surgical videos, named I^2^TM (Fig. 2) to recognize fine-grained triplets (refers to analyzing specific, detailed surgical verbs, instruments, and their corresponding targets).

Our main contributions of this work are summarized as follows:

1. We propose an effective framework I^2^TM by incorporating Intra- and Inter-Triplet Modeling to enhance the accuracy and robustness of surgical video workflow analysis.
2. We present an Intra-TSE in the visual branch, combining both the intra-triplet interaction and the CSTM to enhance visual relational semantics.
3. We present an Inter-Triplet Semantic Enhancer (Inter-TSE) in the textual branch, leveraging both textual and visual graphs to capture the inter-triplet dynamic logic and complementary relationships, thereby enhancing the understanding and reasoning capabilities in surgical video complex scenes.

## Results

### Statistics and official splits of the datasets

**Fig. 3:**
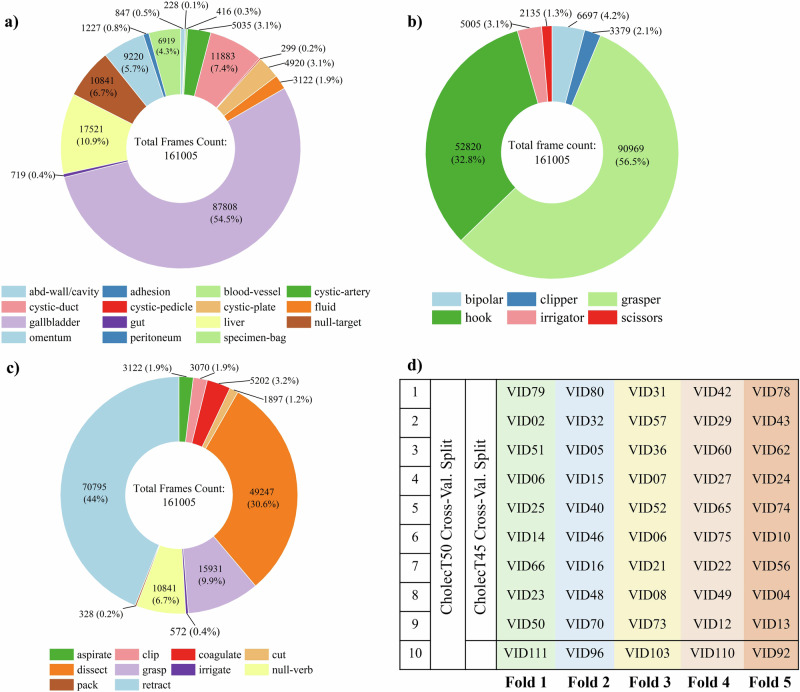
Statistics and official splits of the datasets. **a**–**c** represent the distribution of verb labels, instrument labels, and target labels in the dataset, respectively. **d** is the data split of cross validation on CholecT45 and CholecT50 datasets.

The proportions of verb labels across a total of 161,005 frames are detailed, including labels such as “*aspirate*”, “*cut*” and “*dissect*”, as shown in Fig. 3a. Instrument labels indicate the frequency of each type of instrument used in the dataset, such as “*scissors*”, “*grasper*” and “*hook*” depicted in Fig. 3b. This visualization provides insights into the commonality of different surgical instruments during the procedures. Target labels reveal the occurrence of various targets in the dataset, including anatomical structures like “*abdominal wall/cavity*”, “*adhesion*” and “*cystic artery*” as illustrated in Fig. 3c. These labels point to a challenging and imbalanced multi-label recognition problem. Finally, we report the official cross-validation data split for the CholecT45 and CholecT50 dataset^26^ to highlight the distribution of video IDs across five-fold cross-validation, ensuring a balanced and comprehensive evaluation, as shown in Fig. 3d.

### Validating the I^2^TM method for surgical video workflow analysis

In this section, we compare our proposed approach with state-of-the-art surgical video triplet recognition methods (SOTAs), including Tripnet^15^, Attention Tripnet^17^, RD^17^, RiT^18^, ConceptNet^27^, TD^20^, Forest GCN^19^, CoT^21^, as well as the methods from the CholecTriplet challenge as reported in^28,29^. We maintain consistency with the dataset splitting settings and evaluation metric calculation approaches adopted by the compared methods^26^. To present the comparison results more intuitively, we use more figures in this section, and the corresponding tables are provided in Supplementary Table 1 to Supplementary Table 8.

**Fig. 4:**
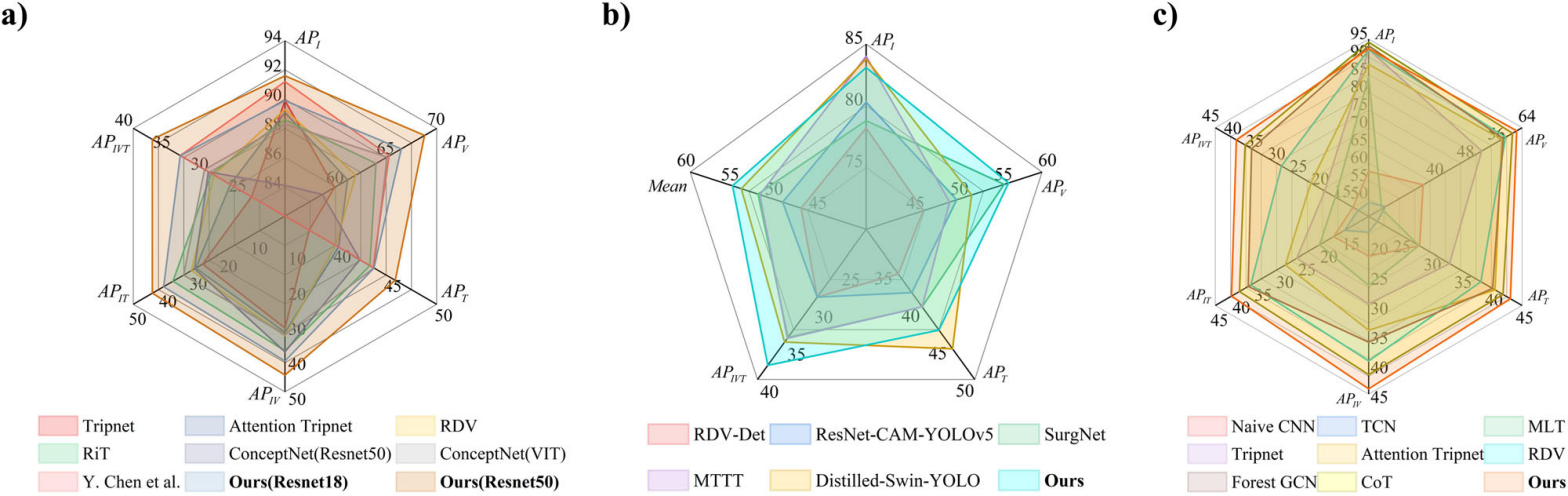
Validating our method for surgical video workflow analysis (Comparison of Component and Association AP). We utilize radar charts to visually compare the performance of our approach against benchmark methods in surgical video triplet recognition, with different methods represented by different colored fill areas. To comprehensively assess the robustness of the methods, we thoroughly consider the three different splits on the surgical triplet dataset (i.e., 5-fold cross-validation split, CholecTriplet Challenge split, and RDV split). **a** shows the comparison results under the 5-fold cross-validation split. **b** presents the comparison results under the CholecTriplet challenge split. **c** shows the comparison results under the RDV split.

Results in Fig. 4a show that our approach outperforms state-of-the-art methods on the main metric *AP*_*IVT*_ in the 5-fold cross-validation test set. For instance, with ResNet18 as the backbone, our approach achieves 4.1% (from 29.7% to 33.8%) gains than the RiT^18^, and with ResNet50 as the backbone, our approach achieves 3.7% (from 33.8% to 37.5%) gains than the TD^20^. Our approach also surpasses competing methods in all of the five remaining AP metrics (i.e., *AP*_*I*_, *AP*_*V*_, *AP*_*T*_, *AP*_*IV*_, and *AP*_*IT*_). In particular, with ResNet18 as the visual backbone, our approach in terms of *AP*_*IV*_ ((increasing from 38.63% to 41.2% compared to RiT^18^) and *AP*_*IT*_ (increasing from 36.9% to 40.0% compared to RiT ^18^)) achieve the largest improvements, indicating that our inter- and intra-triplet enhancement mechanism can generate more accurate predictions of triplet relations.

As shown in Fig. 4b, the evaluation results of the CholecTriplet split highlight the outstanding performance of our approach. Specifically, in terms of *AP*_*V*_ and *AP*_*IVT*_, our approach compared the top submission on the CholecTriplet 2022 challenge^29^, increasing by 4.2% and 2.9% respectively, and achieves the best *AP*_*V*_ and close to the best *AP*_*I*_ and *AP*_*T*_ without introducing additional detectors. Furthermore, as shown in Table 1, we compare our approach with the Top 10 triplet predictions on ChorecTriplet 2021^28^. Due to the large number of classes and high semantic overlap in the triplet classes, we also evaluate and compare the Top K of the proposed model. This metric measures the model’s ability to predict exact triplets within its Top K confidence scores. We report the performance with $K=\left\{5,10,15,20\right\}$, and mean performance of these four thresholds. From the table, it can be seen that our approach has the largest coverage area, especially in the Top 5 where performance is improved from 69.35% to 77.29%, indicating that we have better accuracy in predicting surgical video triplets.

**Table 1:** Top K accuracy of the triplet predictions using CholecTriplet Challenge split

Method	TOP 5	TOP 10	TOP 15	TOP 20	TOP {5:20}
SJTU-IMR	66.50	81.88	84.19	84.89	79.37
SIAT-CAMI	66.58	81.93	88.59	91.84	82.24
HFUT-NUS	65.71	84.18	88.68	90.43	82.25
Digital-Surgery	65.97	81.56	88.78	92.92	82.31
Ceaiik	66.02	81.34	89.74	93.40	82.63
Trequartista	68.50	82.40	88.24	92.29	82.86
Attention-Tripnet	66.86	82.49	91.85	93.25	83.61
HFUT-MedIA	65.05	85.35	91.75	93.59	83.94
Tripnet	67.89	83.99	90.76	93.65	84.07
RDV	69.35	84.38	89.93	93.24	84.23
**I**^**2**^**TM**	**77.29**	**89.78**	**93.17**	**95.59**	**88.96**

Moreover, the comparative results under the RDV split setting are illustrated in Fig. 4c. Compared to the state-of-the-art method, namely CoT^21^, our approach achieved the performance gains of 2%, 2.4%, 1.8% in terms of *AP*_*IVT*_, *AP*_*IV*_, and *AP*_*IT*_, respectively. Considering the above results of the component and association AP comparisons, it is evident that our proposed method effectively acquires more inter-triplet relational semantics, thereby enhancing the accuracy and robustness of recognition in complex surgical scenarios.

**Fig. 5:**
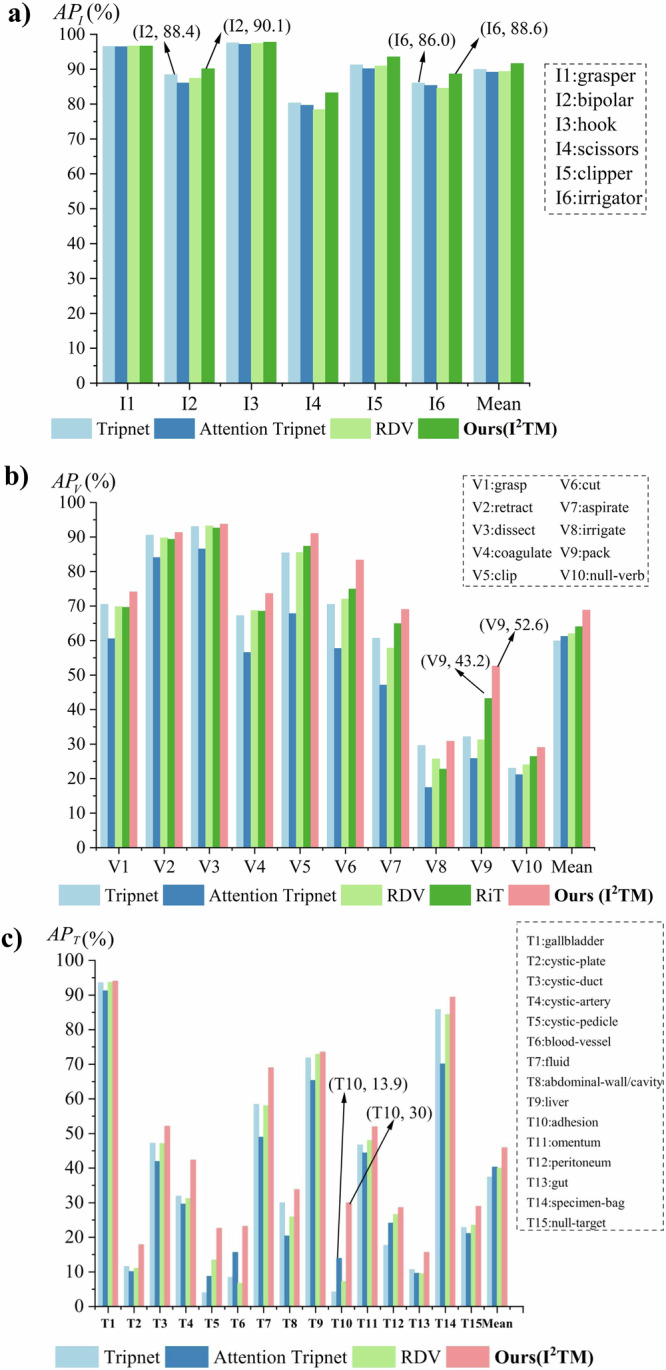
Validating our method for surgical video workflow analysis (Comparison of Class-wise Performances on Component AP). We chose to illustrate the results under the 5-fold cross-validation split. **a**–**c** respectively show the class-wise AP results for instrument, verb, and target in surgical video triplet recognition, respectively.

To explore the benefits of our method in enhancing the recognition, we present the class-wise results obtained from 5-fold cross-validation. Since currently only Tripnet^15^, Attention Tripnet^17^, RDV^17^, and RiT^18^ have reported class-wise results under 5-fold cross-validation split setting, we compare our results solely with those of these methods. The results in the Fig. 5a illustrate the instrument recognition using a 5-fold cross-validation split. In surgical video analysis, instruments are concrete objects with distinct appearances, resulting in generally high recognition accuracy. Particularly, frequently occurring tools such as graspers and hooks exhibit notably high accuracy rates. On the contrary, less commonly appearing instruments like bipolar and irrigator often show lower recognition rates. Our approach in this study effectively improved the recognition rates of bipolar and irrigator instruments after considering intra- and inter-relations within surgical triplets. Specifically, the recognition rate for “bipolar” improves from 88.4% to 90.1%, and for “irrigator”, it improves from 86.0% to 88.6%. This improvement highlights the method’s capability to enhance recognition accuracy across all instrument types, addressing the variability in their appearances in surgical videos.

Figure 5b demonstrates that our proposed approach outperforms previous SOTAs in recognizing various classes of verbs. Specifically, for the recognition of V9: “pack” which comprises only 196 instances in the dataset, our approach achieves an AP of 52.6%, marking an improvement of 9.4% over the best SOTA result. Similarly, the results of target recognition reported in Fig. 5c reveal a significant improvement in recognizing targets with fewer instances, e.g., (“adhesion” and “gut”) compared to predominant categories, e.g., (“gallbladder” and “liver”). Specifically, our approach achieves a significant improvement in T10: “adhesion” recognition, boosting the AP (from 13.9% to 30.0%). We attribute this phenomenon to the robustness of I^2^TM in capturing relational semantics and spatio-temporal reasoning, which helps capture the intra-and inter-triplet mutual relationships.

**Fig. 6:**
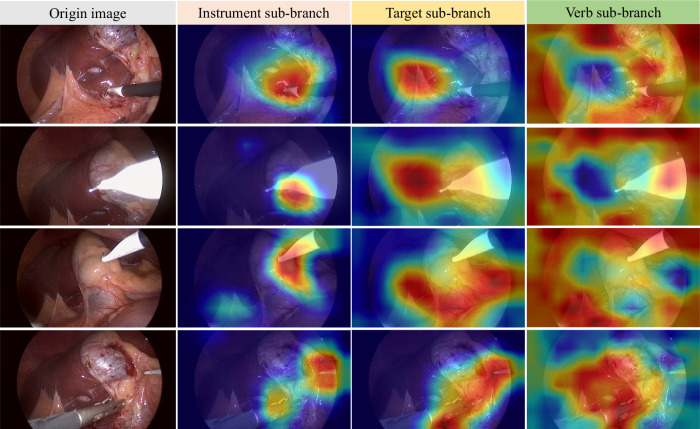
Comparison of attention Maps in instrument, target, and verb sub-branches of the I^2^TM visual branch. The attention maps demonstrate how each sub-branch effectively focuses on its corresponding elements within the surgical context.

Additionally, we randomly select four frames from the video dataset, and present the attention maps of the instrument, target, and verb branches in visual branch of I^2^TM for the chosen images, as shown in the Fig. 6 Compared to the original image, the instrument and target branches effectively focus on the corresponding tools and target regions in the surgery, while the verb branch provides a more comprehensive attention field, facilitating the modeling of both local and global contexts.

**Fig. 7:**
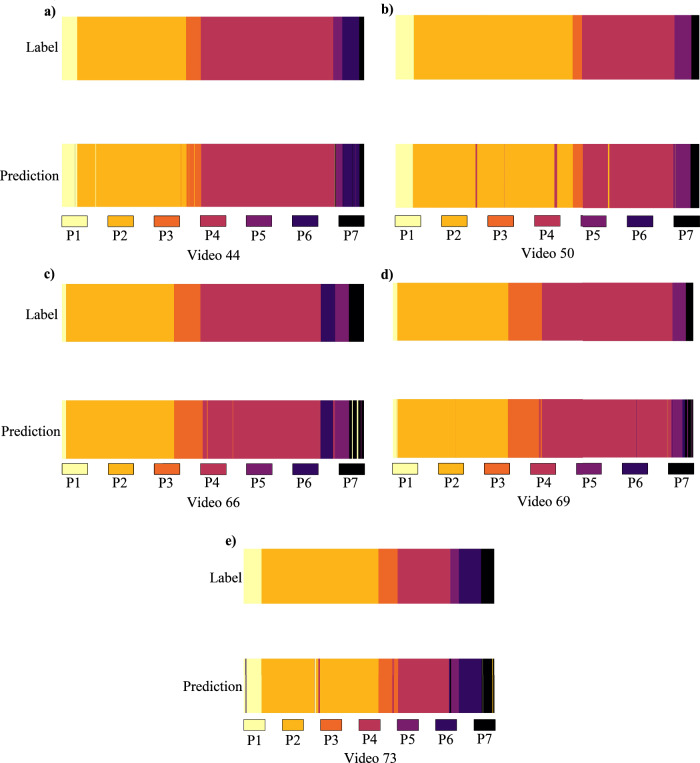
Comparison of ground truth labels and predictions for different phases(P1 to P7) in surgical videos. **a**–**e** represent the visualizedrecognition results for Video 44, Video 50, Video 66, Video 69, and Video 73 in the surgical video phase recognition dataset, respectively.

In this study, we not only validate the effectiveness of the proposed method for surgical video triplet recognition task, but also conduct experiments on single surgical video phase recognition task. As shown in Fig. 7, we randomly select 5 surgical videos, and visualize predictions for different surgical phases (i.e., P1, P2, …, P7) on the 5-fold cross-validation of the surgical video phase recognition dataset^30–32^. Each phase is marked by a specific color. For Video 44 (Fig. 7a) and Video 69 (Fig. 7d), the phases transition smoothly from P1 to P7 with larger segments for P4 in the label, while the prediction matches the overall sequence of phases but shows some noise, particularly in P2, and P3. Video 50 (Fig. 7b) can also predict and identify these phases well, but fragmentation increases, especially during the transition from P1 to P4. In Video 66 (Fig. 7c) and Video 73 (Fig. 7e), long segments of P2, P3, and P6 are observed in the label, along with short segments of P1, P4, P5, and P7. The predictions for P4 and P7 are more fragmented and exhibit frequent transitions between phases. In summary, the proposed approach demonstrates a relatively accurate capability in identifying surgical phases. Although there are some discrepancies in predictions compared to the real situation, it still reflects its potential in surgical video analysis.

### Improving granularity using other techniques

**Fig. 8:**
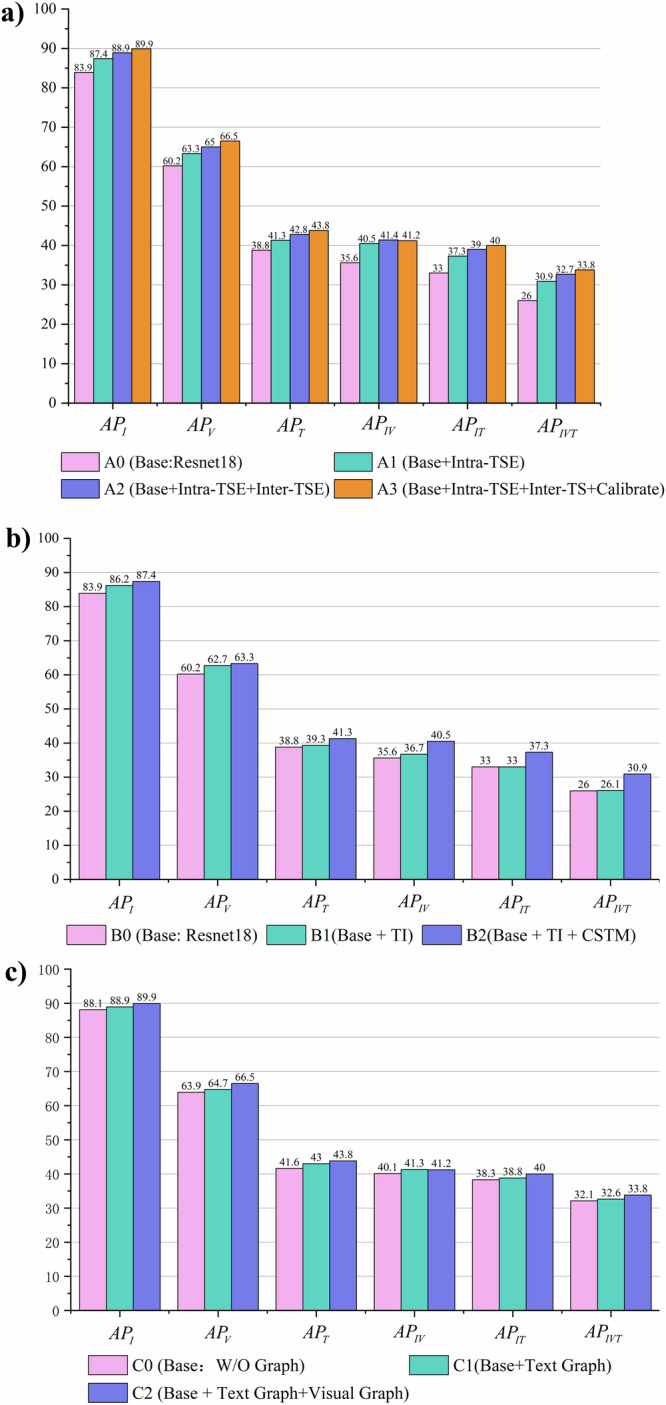
Comparing the performance of techniques for enhancing the granularity of surgical video analysis. **a** Performance improvements with different components of the I^2^TM model. **b** Effectiveness of TI and CSTM integration in Intra-TSE. **c** Impact of textual and visual graphs in Inter-TSE.

In our proposed I^2^TM model, we introduce some special technical modules for medical video analysis. We conduct an ablation study to validate the superiority of the main components in I^2^TM. Figure 8a shows the performance of I^2^TM with different components. Here, A0 denotes that it only uses Resnet18 as the base model for medical video analysis. Compared with A0, the performance of A1 (i.e., base+Intra-TSE) is significantly improved, namely an improvement of 4.4% in *AP*_*IVT*_. It proves that Intra-TSE can effectively enhance the performance of surgical triplet recognition by prompting visual relation-semantic. Compared with A1, the performance gain of A2 (i.e., A1+Inter-TSE) continues to increase when equipped with Inter-TSE, i.e., an improvement of 1.8% in *AP*_*IVT*_, validating the effectiveness of enhanced textual relational semantics. Additionally, the experimental results of A3 (A2 + calibrate) also demonstrate the effectiveness of calibration. To sum up, all components in I^2^TM can jointly improve the performance.

Then we conduct an ablation study to validate the effectiveness of the main components in Intra-TSE. Figure 8b shows the performance of Intra-TSE with different components. Here, B0 denotes it uses ResNet18 as the base model, while B1 (i.e., B0+triplet interaction (TI)) and B2 (i.e., B0+CSTM) denote the integration of TI and CSTM onto the base model, respectively. Compared to the results of B0, both B1 and B2 achieve performance improvements. Specifically, compared to B0, B2 gains 4.8% improvements in *AP*_*IVT*_. This indicates that integrating TI and CSTM in our Intra-TSE effectively enhances intra-triplet relational semantics in both spatial and temporal dimensions, thereby boosting performance in surgical video analysis.

Finally, we conduct an ablation study to validate the importance of the textual graph and visual graph in Inter-TSE. The results are shown in Fig. 8c. Here, C0 denotes the absence of utilizing textual graph and visual graph in I^2^TM, C1 denotes only the textual graph used, and C2 (i.e., Inter-TSE) denotes both the visual graph and the textual graph used. Compared to C0, C1 achieves performance improvement by leveraging a text graph. Results from C2 demonstrate that the joint using of textual and visual graphs yields the best performance, e.g., achieving an improvement of 1.2% over that of C1 in terms of *AP*_*IVT*_. The best performance achieved when both textual and visual graphs are simultaneously used. It is demonstrated that the Inter-TSE in I^2^TM effectively enhances the semantic relationships conveyed by textual prompts, leading to better acquisition of inter-triplet relationships.

## Discussion

To further enhance the performance of visual language models in surgical video analysis, we propose I^2^TM, an effective framework that incorporates intra- and inter-triplet modeling to improve the accuracy and robustness of surgical video analysis. Specifically, we introduce a TSE, consisting of Intra-TSE from the visual branch and Inter-TSE from the textual branch. Specifically, Intra-TSE aims to enhance spatio-temporal relational semantic reasoning by leveraging triplet interaction and introducing the CSTM. Inter-TSE aims to fully capture the semantic relationships among triplets by utilizing visual and textual graphs. Extensive experiments on datasets demonstrate its superior performance. The proposed TSE can also be flexibly integrated into existing visual language models (VLMs), enhancing relational semantics through prompt learning to achieve high-quality visual-text alignment.

We believe that the potential of visual-language models for surgical video analysis is remarkable, as it can significantly enhance real-time monitoring and decision-making during surgery, effectively evolving existing paradigms of surgical operation recording and analysis^33,34^. By combining visual and linguistic information, we can more accurately identify and understand critical steps, anomalies, and potential risks in surgeries. This capability assists surgeons in making more informed decisions during operations, thereby improving the safety and success rates of surgeries^35–39^.

Our research not only aids doctors in real-time surgical decision-making, but also provides detailed reviews and analyses of surgical procedures postoperatively. For example, when complications arise post-surgery, analysis based on visual language models can help doctors retrospectively examine the surgical process to identify possible causes and improve future surgical strategies. Moreover, this technology can be utilized in the training of surgeons, offering detailed step-by-step surgical analysis and key point reminders, thereby helping novice surgeons quickly acquire complex surgical skills. Additionally, the application prospects of surgical video analysis based on visual language models for optimizing medical resources are significant. By automatically analyzing large volumes of surgical videos, we can extract best practices and apply them to the formulation of surgical guidelines and standardized operating procedures. This not only helps to improve the consistency and quality of medical services but also reduces the wastage of medical resources.

**Fig. 9:**
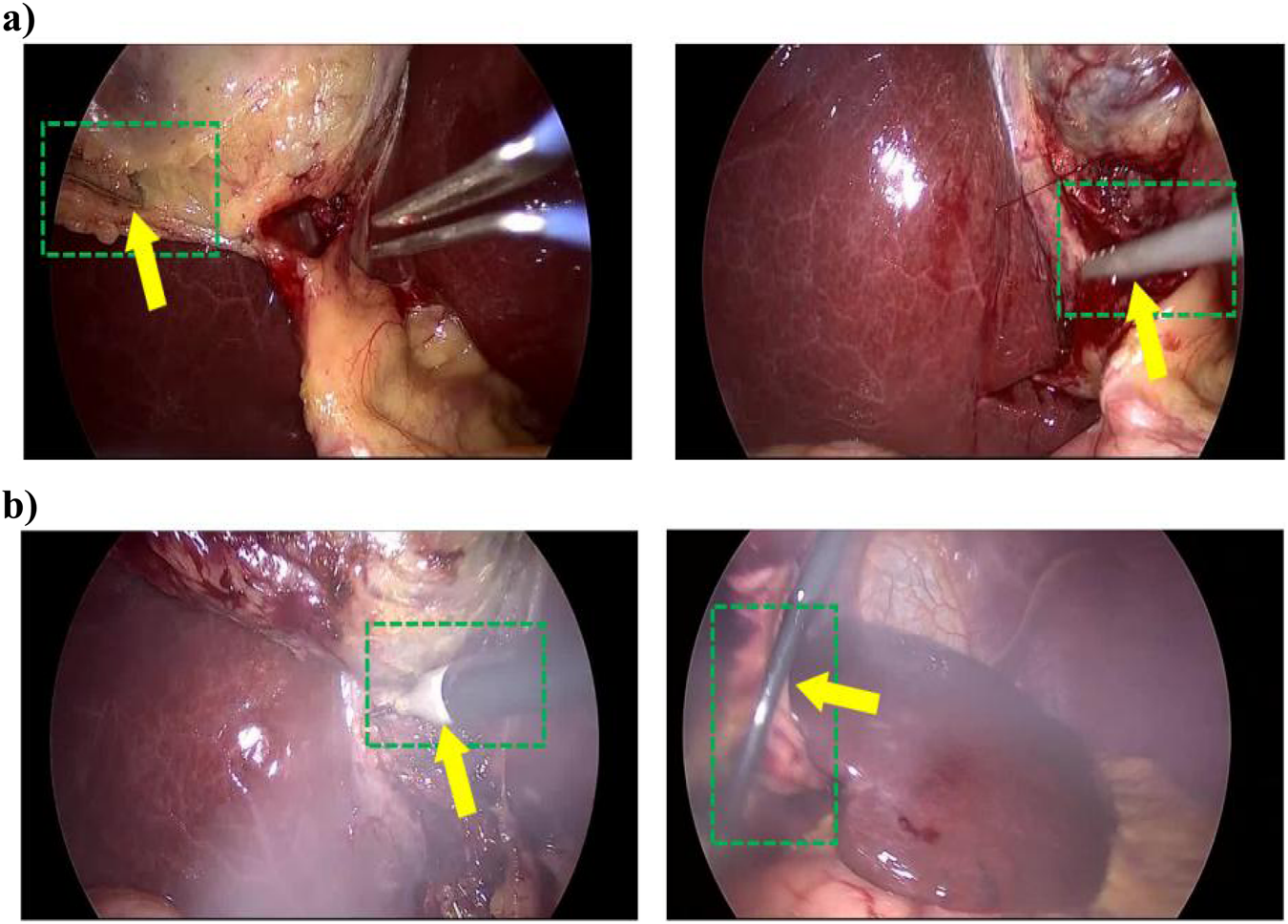
Examples of failure cases using for triplet action recognition. **a** Demonstrate the difficulty in analyzing surgical videos due to surgical tools being located at the edge of the image, and **b** demonstrate the noise interference caused by thick smoke generated during the surgical process on surgical video analysis.

Despite our approach showing good performance superiority and application prospect in medical video analysis, our model still has some limitations. One of the limitations of our surgical video analysis, as illustrated in the Fig. 9a, is that the tools are located at the edges of the image. This results in some failure cases where our model fails to detect the instruments correctly. As shown in the Fig. 9a, our approach for analyzing video content often exhibits poor detection results for tools indicated by the yellow arrows in the green box, primarily due to their placement at the edges of the image. To address this challenge, future research needs to focus on addressing boundary issues to ensure accurate detection and analysis of tools and critical surgical actions at the periphery of the image, thereby improving the accuracy of surgical video analysis.

Another common scenario affecting surgical video analysis is illustrated in the Fig. 9b, where heavy smoke generated during surgery hinders effective video analysis. The area pointed to by the yellow arrow in the green box in the Fig. 9b suffers from significant noise caused by smoke, making accurate analysis difficult. To overcome this issue, we plan to integrate techniques from low-level tasks in computer vision, such as image denoising and dehazing, in our future work. This approach aims to enhance clarity in medical surgical video data before analysis.

Furthermore, the method proposed in this paper has certain limitations due to the text prompts encoded by the text branch being manually constructed (embedded label classes based on specific templates). This approach cannot deeply explore the effects of complex prior knowledge present in surgical videos. Therefore, future work will focus on improving performance by incorporating some a priori knowledge of the task, such as that contained in ontologies^40^ or written materials^41,42^. In addition, the development of more suitable visual language models for surgical video analysis will be explored, including more comprehensive and flexible text prompt methods, and richer embeddings of textual prior knowledge^43–45^.

Additionally, as shown in Supplementary Table 7, we provide the inference speed data of the proposed algorithm. The method presented in this paper still has certain limitations in real-time inference. We are willing to explore more efficient and lightweight approaches in future work to enhance the inference speed, making it more suitable for practical use in real-time surgical video analysis.

## Methods

### Preliminaries

Given a surgical video dataset with *N* sample pairs, i.e., ${{{\mathcal{D}}}}_{train}=\left\{\left({x}_{1},{y}_{1}\right),\ldots ,\left({x}_{N},{y}_{N}\right)\right\}$, where ${x}_{i}\in {{\mathbb{R}}}^{T\times H\times W\times 3}$ refers to the input video clips, we create video clips from *T* frames following ${x}_{i}=\left[{f}_{t-T+1},...,{f}_{t}\right]$. Moreover, the input pipeline is causal as the model learns to predict label *y*_*i*_ from *x*_*i*_ with no access to future frames. Each set of labels of triplet classes can be denoted as ${y}_{i}=\left\{{{y}_{i}}^{I},{{y}_{i}}^{V},{{y}_{i}}^{T},{{y}_{i}}^{IVT}\right\}$, where *I*, *V*, *T* and *IVT* are denoted as *instrument*, *verb*, *target*, and *<instrument, verb, target>*, respectively. Our objective of surgical video workflow analysis is to train a classification model with minimized prediction error on the test dataset.

### Intra-Triplet Semantic Enhancer (Intra-TSE)

In the surgical triplet<*instrument, verb, target*>, uncovering potential intra-triplet relationships is crucial for accurate triplet recognition. Therefore, we perform cross-component triplet interaction (TI) (Supplementary Fig. 3) using the features extracted by the vision backbone with instrument features as the interacting mediator, thus obtaining potential triplet relationships. Specifically, we first use an MLP to enhance the semantic information of the instrument sub-branch features for triplet interaction, as follows: $\hat{{f}_{{{\rm{i}}}}}={{\rm{MLP}}}({f}_{{{\rm{i}}}})$, *f*_i_ = *f*(*x*_*i*_), where *f*( ⋅ ) represents the feature extraction using the visual backbone. Therefore, the next process can be defined as:

1 $$\begin{array}{ll}\hat{{{\bf{Q}}}},\hat{{{\bf{K}}}},\hat{{{\bf{V}}}}={{\rm{R}}}\left({{{\rm{Dconv}}}}_{{{\bf{Q}}}}\left({{{\rm{Conv}}}}_{{{\bf{Q}}}}({{\bf{Q}}})\right)\right),{{\rm{R}}}\left({{{\rm{Dconv}}}}_{{{\bf{K}}}}\left({{{\rm{Conv}}}}_{{{\bf{K}}}}({{\bf{K}}})\right)\right),\\ \qquad\qquad\quad{{\rm{R}}}\left({{{\rm{Dconv}}}}_{{{\bf{V}}}}\left({{{\rm{Conv}}}}_{{{\bf{V}}}}({{\bf{V}}})\right)\right),\end{array}$$

where the input **Q** comes from a different component than **V** and **K**. Conv_**V,K,Q**_( ⋅ ), Dconv_**V,K,Q**_( ⋅ ), and R( ⋅ ) denote 1 × 1 convolution, 3 × 3 depth-wise convolution and reshape operation, respectively. Then, we obtain the results of the interaction between the verb sub-branch feature *f*_v*&*i_ and the target sub-branch feature with the instrument feature *f*_t*&*i_, as follows:

2 $${{{\mathbf{\xi }}}}_{{{\rm{ca}}}}({{\bf{V}}},{{\bf{K}}},{{\bf{Q}}})={{\rm{Con}}}{{{\rm{v}}}}_{{{\rm{out}}}}({{\rm{R}}}({{\rm{Softmax}}}({\hat{{{\bf{K}}}}}^{{{\bf{T}}}}\hat{{{\bf{Q}}}})\hat{{{\bf{V}}}})),$$

3 $${f}_{{{\rm{v}}}\& {{\rm{i}}}}={{{\mathbf{\xi }}}}_{{{\rm{ca}}}}({{\bf{V}}}={{\rm{LN}}}(\hat{{f}_{i}}),{{\bf{K}}}={{\rm{LN}}}(\hat{{f}_{i}}),{{\bf{Q}}}={{\rm{LN}}}({f}_{v})),$$

4 $${f}_{{{\rm{t}}}\& {{\rm{i}}}}={{{\mathbf{\xi }}}}_{{{\rm{ca}}}}({{\bf{V}}}={{\rm{LN}}}(\hat{{f}_{i}}),{{\bf{K}}}={{\rm{LN}}}(\hat{{f}_{i}}),{{\bf{Q}}}={{\rm{LN}}}({f}_{t})),$$

where LN denotes the LayerNorm function^46^, as well a ${f}_{{{\rm{v}}}}\in {{\mathbb{R}}}^{H\times W\times C}$, and ${f}_{{{\rm{t}}}}\in {{\mathbb{R}}}^{H\times W\times C}$ are input features of the verb branch and the target branch, respectively. Furthermore, considering that the verb in surgery represents the interaction process between the instrument and the target, a dual cross-attention mechanism^47,48^ is employed in the verb branch to facilitate the acquisition of the collaborative interaction results between the verb, instrument, and target, as below:

5 $${f}_{{{\rm{v}}}\& {{\rm{t}}}}={\xi }_{{{\rm{ca}}}}({{\bf{V}}}={{\rm{LN}}}({\hat{f}}_{{{\rm{t}}}}),{{\bf{K}}}={{\rm{LN}}}({\hat{f}}_{{{\rm{t}}}}),{{\bf{Q}}}={{\rm{LN}}}({f}_{{{\rm{v}}}\& {{\rm{i}}}})),$$

6 $${\hat{f}}_{{{\rm{v}}}}={{\rm{FFN}}}({f}_{{{\rm{v}}}\& {{\rm{t}}}}+{f}_{{{\rm{v}}}\& {{\rm{i}}}}+{f}_{{{\rm{v}}}})+({f}_{{{\rm{v}}}\& {{\rm{t}}}}+{f}_{{{\rm{v}}}\& {{\rm{i}}}}+{f}_{{{\rm{v}}}}),$$

7 $${\hat{f}}_{{{\rm{t}}}}={{\rm{FFN}}}({f}_{{{\rm{t}}}\& {{\rm{i}}}}+{f}_{{{\rm{t}}}})+({f}_{{{\rm{t}}}\& {{\rm{i}}}}+{f}_{{{\rm{t}}}}),$$

where FFN denotes Feed-Forward Network. Finally, after undergoing triplet interaction, we obtain the outputs $\hat{{f}_{{{\rm{v}}}}}$, $\hat{{f}_{{{\rm{i}}}}}$, $\hat{{f}_{{{\rm{t}}}}}$ of the verb, instrument, and target sub-branches, respectively.

Through the TI, the visual relational semantics of all component branches intra-triplet have received initial spatial enhancement. To better explore potential representations of visual semantics^49,50^, we utilize bidirectional LSTM (BiLSTM)^51,52^ to model the sequence of features obtained along the horizontal and vertical directions. Then, considering the temporal relationship characteristics of surgical videos, we combine the temporal transformer to present CSTM (Supplementary Fig. 1).

For an input ${{\bf{I}}}\in {{\mathbb{R}}}^{H\times W\times C},{\left\{{{\bf{I}}}[:,i,:]\in {{\mathbb{R}}}^{H\times C}\right\}}_{i = 1}^{W}$ is viewed as a set of sequences, where *H* denotes the height of the input features, serving as the number of tokens in the vertical direction, *W* represents the width of the input features, indicating the number of sequences in the horizontal direction, and *C* is the channel dimension. All sequences **I**[: , *i*, : ] are input into the vertical BiLSTM with shared weights and hidden dimension *D* : ${{{\bf{S}}}}^{{{\rm{ver}}}}[:,i,:]={\mathrm{BiLSTM}}\,\left({{\bf{I}}}[:,i,:]\right)$

Similarly, we obtain the horizontal direction: ${{{\bf{S}}}}^{{{\rm{hor}}}}[j,:,:]={\mathrm{BiLSTM}}\,\left({{\bf{I}}}[j,:,:]\right)$. Then, we combine ${\left\{{{{\bf{S}}}}^{{{\rm{ver}}}}[:,i,:]\right\}}_{i = 1}^{W}$ into **S**^ver^ and ${\left\{{{{\bf{S}}}}^{{{\rm{hor}}}}[j,:,:]\right\}}_{i = 1}^{H}$ into **S**^hor^. Next, we concatenate the obtained **S**^ver^ and **S**^hor^, then perform a pooling operation and project through fully connected layers before finally inputting into the Temporal transformer. These processes can be expressed as follows:

8 $$\left\{\begin{array}{l}{{\bf{S}}}={{\rm{Cat}}}\left({{{\bf{S}}}}^{{{\rm{ver}}}},{{{\bf{S}}}}^{{{\rm{hor}}}}\right),\quad \\ \hat{{{\bf{I}}}}={{\rm{T}}}\,{\mbox{-}}\,{{\rm{trans}}}({{\rm{FC}}}({{\rm{GAP}}}({{\bf{S}}}))),\quad \end{array}\right.$$

where Cat, GAP, FC, and T-trans respectively represent Contact, Global Average Pooling, Fully Connected Layer, and Temporal-transformer. Finally, through CSTM, we obtain the output of Intra-TSE as $\hat{{{\bf{I}}}}\in {{\mathbb{R}}}^{B\times D}$.

### Inter-Triplet Semantic Enhancer (Inter-TSE)

We recognize that the significance of surgical actions extends beyond individual elements, being dynamically defined by the relationships among triplets. Specifically, the action sequences represented by one triplet often logically precede or complement those depicted by others. Therefore, when designing to capture intra-triplet relationships, we also introduce a semantic enhancement module to address inter-triplet relationships.

Considering the popularity and outstanding performance in the computer vision community, we utilize the text encoder from the vision language model CLIP as the textual encoder in this paper^53^. Then, in the triplet surgical video dataset containing *C*_ivt_ possible surgical triplets ${\left\{{l}_{n}^{{{\rm{ivt}}}}\right\}}_{n = 1}^{{C}_{{{\rm{ivt}}}}}$ of <*instrument*, *verb*, *target*>, we pre-define a triplet text prompt template ${p}_{{{\rm{ivt}}}}\left({l}_{n}^{{{\rm{ivt}}}}\right)=$ “*The surgery is currently using a* [*Instrument*] *to* [*Verb*] *the* [*Target*]” for each triplet, where “[*Instrument*]”, “[*Verb*]” and “[*Target*]” are replaced with their corresponding class names in each triplet. Each template is applied with textual encoder to generate triplet text prompt features **T**^ivt^:

9 $${{{\bf{T}}}}^{{{\rm{ivt}}}}={\left\{{{{\bf{t}}}}_{n}^{{{\rm{ivt}}}}\right\}}_{n = 1}^{{C}_{{{\rm{ivt}}}}}\in {{\mathbb{R}}}^{{C}_{{{\rm{ivt}}}}\times D},{\left\{{{{\bf{t}}}}_{n}^{{{\rm{ivt}}}}\right\}}_{n = 1}^{{C}_{{{\rm{ivt}}}}}={\left\{{{\rm{F}}}\left({p}_{{{\rm{ivt}}}}\left({l}_{{{\rm{n}}}}^{{{\rm{ivt}}}}\right)\right)\right\}}_{n = 1}^{{C}_{{{\rm{ivt}}}}},$$

where F( ⋅ ) and *D* denote the textual encoder and the prompt feature dimension, respectively. **T**^ivt^ embodies the global semantic features, aiming to facilitate the inter-triplet interaction to enhance the understanding of the entire surgical video. Similarly, we have also pre-define the instrument text prompt template ${p}_{i}\left({l}_{n}^{i}\right)=$ “*The surgery is currently using a*[*Instrument*]”, verb text prompt template ${p}_{v}\left({l}_{n}^{v}\right)=$ “*The surgery is currently in the process of* [*Verb*]”, and target text prompt template ${p}_{t}\left({l}_{n}^{t}\right)=$ “*The surgery is currently for the*[*Target*]”. Therefore, we can also obtain ${{{\bf{T}}}}^{{{\rm{i}}}}={\left\{{{{\bf{t}}}}_{n}^{{{\rm{i}}}}\right\}}_{n = 1}^{{C}_{{{\rm{i}}}}}\in {{\mathbb{R}}}^{{C}_{{{\rm{i}}}}\times D}$, ${{{\bf{T}}}}^{{{\rm{v}}}}={\left\{{{{\bf{t}}}}_{n}^{{{\rm{v}}}}\right\}}_{n = 1}^{{C}_{{{\rm{v}}}}}\in {{\mathbb{R}}}^{{C}_{{{\rm{v}}}}\times D}$, and ${{{\bf{T}}}}^{{{\rm{t}}}}={\left\{{{{\bf{t}}}}_{n}^{{{\rm{t}}}}\right\}}_{n = 1}^{{C}_{{{\rm{t}}}}}\in {{\mathbb{R}}}^{{C}_{{{\rm{t}}}}\times D}$, where *C*_i_, *C*_v_, and *C*_t_ represent the number of classes for instrument, verb, and target, respectively.

To explore the inter-triplet relational semantics in the textual space, and provide valuable structural semantics for triplet recognition, we construct the textual graph, denoted as:

10 $${{{\mathcal{G}}}}_{{{\rm{text}}}}=\left\{{{{\mathcal{G}}}}_{{{\rm{text}}}}^{{{\rm{i}}}},{{{\mathcal{G}}}}_{{{\rm{text}}}}^{{{\rm{v}}}},{{{\mathcal{G}}}}_{{{\rm{text}}}}^{{{\rm{t}}}},{{{\mathcal{G}}}}_{{{\rm{text}}}}^{{{\rm{ivt}}}}\right\},$$

where ${{{\mathcal{G}}}}_{{{\rm{text}}}}^{{{\rm{i}}}}$, ${{{\mathcal{G}}}}_{{{\rm{text}}}}^{{{\rm{v}}}}$, ${{{\mathcal{G}}}}_{{{\rm{text}}}}^{{{\rm{t}}}}$, and ${{{\mathcal{G}}}}_{{{\rm{text}}}}^{{{\rm{ivt}}}}$ are equal to $\left\{{{{\bf{T}}}}^{{{\rm{i}}}},{{{\bf{A}}}}_{{{\rm{text}}}}^{{{\rm{i}}}}\right\}$, $\left\{{{{\bf{T}}}}^{{{\rm{v}}}},{{{\bf{A}}}}_{{{\rm{text}}}}^{{{\rm{v}}}}\right\}$, $\left\{{{{\bf{T}}}}^{{{\rm{t}}}},{{{\bf{A}}}}_{{{\rm{text}}}}^{{{\rm{t}}}}\right\}$, and $\left\{{{{\bf{T}}}}^{{{\rm{ivt}}}},{{{\bf{A}}}}_{{{\rm{text}}}}^{{{\rm{ivt}}}}\right\}$, respectively. Here, **T**^i^, **T**^v^, **T**^t^, and **T**^ivt^ represent semantic text prompt features representing different classes obtained through text encoder as nodes in the graph, while ${{{\bf{A}}}}_{{{\rm{text}}}}^{{{\rm{i}}}}$, ${{{\bf{A}}}}_{{{\rm{text}}}}^{{{\rm{v}}}}$, ${{{\bf{A}}}}_{{{\rm{text}}}}^{{{\rm{t}}}}$, and ${{{\bf{A}}}}_{{{\rm{text}}}}^{{{\rm{ivt}}}}$ represent the relationships between different nodes. Taking ${{{\mathcal{G}}}}_{{{\rm{text}}}}^{{{\rm{i}}}}$ as an example, where ${{{\bf{T}}}}^{{{\rm{i}}}}={\left\{{{{\bf{t}}}}_{n}^{{{\rm{i}}}}\right\}}_{n = 1}^{{C}_{{{\rm{i}}}}}\in {{\mathbb{R}}}^{{C}_{{{\rm{i}}}}\times D}$, and ${{{\bf{t}}}}_{n}^{{{\rm{i}}}}$ represents the text feature of the *n*^*th*^ category of instruments. Since classification is achieved by computing cosine similarity between features in CLIP, the edge relationship ${{{\bf{A}}}}_{{{\rm{text}}}}^{{{\rm{i}}}}$ between different nodes is characterized by computing cosine similarity between different nodes and can be derived as:

11 $${{{\bf{A}}}}_{{{\rm{text}}}}^{{{\rm{i}}}}={\left\{{({{{\rm{a}}}}_{{{\rm{text}}}}^{{{\rm{i}}}})}^{m,n}\right\}}_{m = 1,n = 1}^{{C}_{{{\rm{i}}}}},{({{{\rm{a}}}}_{{{\rm{text}}}}^{{{\rm{i}}}})}^{m,n}={{\rm{sim}}}\left({{{\rm{t}}}}_{m}^{{{\rm{i}}}},{{{\rm{t}}}}_{n}^{{{\rm{i}}}}\right),$$

where ${({{{\rm{a}}}}_{{{\rm{text}}}}^{{{\rm{i}}}})}^{m,n}$ represents the edge relationship *m*th and *n*th textual graph nodes, and ${{\rm{sim}}}\left(\cdot ,\cdot \right)$ denotes the function of cosine similarity.

In contrast to textual features, the visual feature map represents the global visual features of the image as a whole rather than representing features for each category. Therefore, each visual feature cannot be directly used to construct the visual graph. To address this issue, we first leverage prototype learning to initialize the representation of the visual feature of each category. Specifically, we aggregate and take the average of the features obtained through the vision encoder for each category, ultimately obtaining the global features:

12 $$\begin{array}{r}{{{\bf{V}}}}^{{{\rm{i}}}},{{{\bf{V}}}}^{{{\rm{v}}}},{{{\bf{V}}}}^{{{\rm{t}}}},{{{\bf{V}}}}^{{{\rm{ivt}}}}={\left\{{{{\bf{v}}}}_{n}^{{{\rm{i}}}}\right\}}_{n = 1}^{{C}_{{{\rm{i}}}}}\in {{\mathbb{R}}}^{{C}_{{{\rm{i}}}}\times D},{\left\{{{{\bf{v}}}}_{n}^{{{\rm{v}}}}\right\}}_{n = 1}^{{C}_{{{\rm{v}}}}}\in {{\mathbb{R}}}^{{C}_{{{\rm{v}}}}\times D},\\ {\left\{{{{\bf{v}}}}_{n}^{{{\rm{t}}}}\right\}}_{n = 1}^{{C}_{{{\rm{t}}}}}\in {{\mathbb{R}}}^{{C}_{{{\rm{t}}}}\times D},{\left\{{{{\bf{v}}}}_{n}^{{{\rm{v}}}}\right\}}_{n = 1}^{{C}_{{{\rm{ivt}}}}}\in {{\mathbb{R}}}^{{C}_{{{\rm{ivt}}}}\times D}.\end{array}$$

The above results are from video frames ${\left\{{f}_{n}^{{{\rm{i}}}}\right\}}_{n = 1}^{{C}_{{{\rm{i}}}}}$,${\left\{{f}_{n}^{{{\rm{v}}}}\right\}}_{n = 1}^{{C}_{{{\rm{v}}}}}$,${\left\{{f}_{n}^{{{\rm{t}}}}\right\}}_{n = 1}^{{C}_{{{\rm{t}}}}}$, ${\left\{{f}_{n}^{{{\rm{ivt}}}}\right\}}_{n = 1}^{{C}_{{{\rm{ivt}}}}}$ (only in the training set) belonging to each class are extracted as prototypes for all categories. Then, these prototypes can be used to construct the visual graph, and set to be learnable during training.

Similarly, we utilize the output of visual encoder to build the visual graph, denoted as : ${{{\mathcal{G}}}}_{{{\rm{vision}}}}=\left\{{{{\mathcal{G}}}}_{{{\rm{vision}}}}^{{{\rm{i}}}},{{{\mathcal{G}}}}_{{{\rm{vision}}}}^{{{\rm{v}}}},{{{\mathcal{G}}}}_{{{\rm{vision}}}}^{{{\rm{t}}}},{{{\mathcal{G}}}}_{{{\rm{vision}}}}^{{{\rm{ivt}}}}\right\}$, where ${{{\mathcal{G}}}}_{{{\rm{vision}}}}^{{{\rm{i}}}}$, ${{{\mathcal{G}}}}_{{{\rm{vision}}}}^{{{\rm{v}}}}$, ${{{\mathcal{G}}}}_{{{\rm{vision}}}}^{{{\rm{t}}}}$, and ${{{\mathcal{G}}}}_{{{\rm{vision}}}}^{{{\rm{ivt}}}}$ are equal to $\left\{{{{\bf{V}}}}^{{{\rm{i}}}},{{{\bf{A}}}}_{{{\rm{vision}}}}^{{{\rm{i}}}}\right\}$, $\left\{{{{\bf{V}}}}^{{{\rm{v}}}},{{{\bf{A}}}}_{{{\rm{vision}}}}^{{{\rm{v}}}}\right\}$, $\left\{{{{\bf{V}}}}^{{{\rm{t}}}},{{{\bf{A}}}}_{{{\rm{vision}}}}^{{{\rm{t}}}}\right\}$, and $\left\{{{{\bf{V}}}}^{{{\rm{ivt}}}},{{{\bf{A}}}}_{{{\rm{vision}}}}^{{{\rm{ivt}}}}\right\}$, respectively. Specifically, taking ${{{\bf{A}}}}_{{{\rm{vision}}}}^{{{\rm{i}}}}={\left\{{({{{\rm{a}}}}_{{{\rm{vision}}}}^{{{\rm{i}}}})}^{m,n}\right\}}_{m = 1,n = 1}^{{C}_{{{\rm{i}}}}}$ as an example, its edge relationships are obtained by calculating the cosine similarity between nodes in the visual graph.

Notably, in previous works addressing surgical video workflow analysis using graphs, only a single modality (i.e., textual modality^19,27^) was employed to capture semantics, lacking the utilization of cross-modal semantics between textual and visual. Therefore, after constructing the dual-modal graph ${{\mathcal{G}}}=\left\{{{{\mathcal{G}}}}_{{{\rm{text}}}},{{{\mathcal{G}}}}_{{{\rm{vision}}}}\right\}$, structural semantics from both textual and visual graphs are adaptively introduced^54,55^. Specifically, focusing on the instrument branch as an example, when given the textual features t^i^ from the textual encoder of CLIP, we treat t^i^ as a node and connect separately with the textual/visual graphs to construct a new extended graph then the edges relations between different nodes are computed as:

13 $$\begin{array}{c}{{\bf{T}}}{{{\bf{T}}}}^{{{\rm{i}}}}=[{{{\rm{t}}}}^{{{\rm{i}}}},{{{\bf{T}}}}^{{{\rm{i}}}}],{{\bf{T}}}{{{\bf{V}}}}^{{{\rm{i}}}}=[{{{\rm{t}}}}^{{{\rm{i}}}},{{{\bf{V}}}}^{{{\rm{i}}}}],\\ {{{\bf{A}}}}_{{{\rm{t}}}\leftrightarrow {{\rm{t}}}}^{{{\rm{i}}}}=\left[\begin{array}{cc}1&{{\rm{sim}}}\left({{{\rm{t}}}}^{{{\rm{i}}}},{{{\bf{T}}}}^{{{\rm{i}}}}\right)\\ {{\rm{sim}}}\left({{{\bf{T}}}}^{{{\rm{i}}}},{{{\rm{t}}}}^{{{\rm{i}}}}\right)&{{{\bf{A}}}}_{{{\rm{text}}}}^{{{\rm{i}}}}\end{array}\right],\\ {{{\bf{A}}}}_{{{\rm{t}}}\leftrightarrow {{\rm{v}}}}^{{{\rm{i}}}}=\left[\begin{array}{cc}1&{{\rm{sim}}}\left({{{\rm{t}}}}^{{{\rm{i}}}},{{{\bf{V}}}}^{{{\rm{i}}}}\right)\\ {{\rm{sim}}}\left({{{\bf{V}}}}^{{{\rm{i}}}},{{{\rm{t}}}}^{{{\rm{i}}}}\right)&{{{\bf{A}}}}_{{{\rm{vision}}}}^{{{\rm{i}}}}\end{array}\right]\end{array}$$

Following the above method, we can integrate and embed triplet textual features into the dual graph space, laying the foundation for interaction and relational semantic transfer between textual features and the dual graph. Subsequently, relational semantics are excavated from each node in the textual and visual graphs using the GCN ${\Phi }_{{{\rm{t}}}\leftrightarrow {{\rm{t}}}}^{{{\rm{i}}}}$ and ${\Phi }_{{{\rm{t}}}\leftrightarrow {{\rm{v}}}}^{{{\rm{i}}}}$ get:

14 $$\begin{array}{c}\hat{{{\bf{T}}}{{{\bf{T}}}}^{{{\rm{i}}}}}={\Phi }_{{{\rm{t}}}\leftrightarrow {{\rm{t}}}}^{{{\rm{i}}}}\left({{\bf{T}}}{{{\bf{T}}}}^{{{\rm{i}}}},{{\hat{{{\bf{A}}}}}^{{{\rm{i}}}}}_{{{\rm{t}}}\leftrightarrow {{\rm{t}}}}\right)=\rho \left({{\hat{{{\bf{A}}}}}^{{{\rm{i}}}}}_{{{\rm{t}}}\leftrightarrow {{\rm{t}}}}{{\bf{T}}}{{{\bf{T}}}}^{{{\rm{i}}}}{{{{\bf{W}}}}^{{{\rm{i}}}}}_{{{\rm{t}}}\leftrightarrow {{\rm{t}}}}\right),\\ \hat{{{\bf{T}}}{{{\bf{V}}}}^{{{\rm{i}}}}}={\Phi }_{{{\rm{t}}}\leftrightarrow v}^{{{\rm{i}}}}\left({{\bf{T}}}{{{\bf{V}}}}^{{{\rm{i}}}},{{\hat{{{\bf{A}}}}}^{{{\rm{i}}}}}_{{{\rm{t}}}\leftrightarrow {{\rm{v}}}}\right)=\rho \left({{\hat{{{\bf{A}}}}}^{{{\rm{i}}}}}_{{{\rm{t}}}\to {{\rm{v}}}}{{\bf{T}}}{{{\bf{V}}}}^{{{\rm{i}}}}{{{{\bf{W}}}}^{{{\rm{i}}}}}_{{{\rm{t}}}\leftrightarrow {{\rm{v}}}}\right),\end{array}$$

where ${{\hat{{{\bf{A}}}}}^{{{\rm{i}}}}}_{{{\rm{t}}}\leftrightarrow {{\rm{t}}}}={{{{\bf{D}}}}^{{{\rm{i}}}}}_{{{\rm{t}}}\leftrightarrow {{\rm{t}}}}{{{{\bf{A}}}}^{{{\rm{i}}}}}_{{{\rm{t}}}\leftrightarrow {{\rm{t}}}}{{{{\bf{D}}}}^{{{\rm{i}}}}}_{{{\rm{t}}}\leftrightarrow {{\rm{t}}}}$ and ${{\hat{{{\bf{A}}}}}^{{{\rm{i}}}}}_{{{\rm{t}}}\leftrightarrow {{\rm{v}}}}={{{{\bf{D}}}}^{{{\rm{i}}}}}_{{{\rm{t}}}\leftrightarrow {{\rm{v}}}}{{{{\bf{A}}}}^{{{\rm{i}}}}}_{{{\rm{t}}}\leftrightarrow {{\rm{v}}}}{{{{\bf{D}}}}^{{{\rm{i}}}}}_{{{\rm{t}}}\leftrightarrow {{\rm{v}}}}$ are the adjacent matrix for graph learning. *ρ* is a non-linear function. And the ${{{{\bf{D}}}}^{{{\rm{i}}}}}_{{{\rm{t}}}\leftrightarrow {{\rm{v}}}}$ and ${{{{\bf{D}}}}^{{{\rm{i}}}}}_{{{\rm{t}}}\leftrightarrow {{\rm{v}}}}$ are the matrices used for Laplace normalization for the edges as:

15 $${{{{\bf{D}}}}^{{{\rm{i}}}}}_{{{\rm{t}}}\leftrightarrow {{\rm{t}}}}={\mathrm{diag}}\,\left(\sum\limits_{p=1}^{{C}_{{{\rm{i}}}}}{\left({{{{\bf{A}}}}^{{{\rm{i}}}}}_{{{\rm{t}}}\leftrightarrow {{\rm{t}}}}+{{\bf{I}}}\right)}_{p}\right),{{{{\bf{D}}}}^{{{\rm{i}}}}}_{{{\rm{t}}}\leftrightarrow {{\rm{v}}}}={\mathrm{diag}}\,\left(\sum\limits_{p=1}^{{C}_{{{\rm{i}}}}}{\left({{{{\bf{A}}}}^{{{\rm{i}}}}}_{{{\rm{t}}}\leftrightarrow {{\rm{v}}}}+{{\bf{I}}}\right)}_{p}\right).$$

Based on Eq. (14) above, we seek the semantics of other nodes, i.e., inter-triplet, in the graph to refine our features based on the relations. (i.e., adjacency matrix). Therefore, we can obtain refined features as ${{\bf{t}}}{{{\bf{t}}}}^{{{\rm{i}}}}=\hat{{{\bf{T}}}{{{\bf{T}}}}^{{{\rm{i}}}}}[0,:]$ and ${{\bf{t}}}{{{\bf{v}}}}^{{{\rm{i}}}}=\hat{{{\bf{T}}}{{{\bf{V}}}}^{{{\rm{i}}}}}[0,:]$. The final output is : ${{{\bf{G}}}}^{{{\rm{i}}}}=({{\bf{t}}}{{{\bf{t}}}}^{{{\rm{i}}}}+{{\bf{t}}}{{{\bf{v}}}}^{{{\rm{i}}}}+{{{\bf{t}}}}^{{{\rm{i}}}})\in {{\mathbb{R}}}^{{C}_{{{\rm{i}}}}\times D}$. Similarly, we obtain the outputs of the verb, target, and triplet sub-branches in the cross-modality graph as ${{{\bf{G}}}}^{{{\rm{v}}}}\in {{\mathbb{R}}}^{{C}_{{{\rm{v}}}}\times D}$, ${{{\bf{G}}}}^{{{\rm{t}}}}\in {{\mathbb{R}}}^{{C}_{{{\rm{t}}}}\times D}$, and ${{{\bf{G}}}}^{{{\rm{ivt}}}}\in {{\mathbb{R}}}^{{C}_{{{\rm{ivt}}}}\times D}$ respectively.

### Prediction calibration

After obtaining the Intra-TSE representation ${\hat{{{\bf{I}}}}}^{{{\rm{i}}}}\in {{\mathbb{R}}}^{B\times D}$, ${\hat{{{\bf{I}}}}}^{{{\rm{v}}}}\in {{\mathbb{R}}}^{B\times D}$, ${\hat{{{\bf{I}}}}}^{{{\rm{t}}}}\in {{\mathbb{R}}}^{B\times D}$, and ${\hat{{{\bf{I}}}}}^{{{\rm{ivt}}}}\in {{\mathbb{R}}}^{B\times D}$, the Inter-TSE representation ${{{\bf{G}}}}^{{{\rm{i}}}}\in {{\mathbb{R}}}^{{C}_{{{\rm{i}}}}\times D}$,${{{\bf{G}}}}^{{{\rm{v}}}}\in {{\mathbb{R}}}^{{C}_{{{\rm{v}}}}\times D}$, ${{{\bf{G}}}}^{{{\rm{t}}}}\in {{\mathbb{R}}}^{{C}_{{{\rm{t}}}}\times D}$, and ${{{\bf{G}}}}^{{{\rm{ivt}}}}\in {{\mathbb{R}}}^{{C}_{{{\rm{ivt}}}}\times D}$, the predict logits are can be using matrix multiplication between them as follow:

16 $$\left\{\begin{array}{l}{P}^{{{\rm{i}}}}={\hat{{{\bf{I}}}}}^{{{\rm{i}}}}{{{{\rm{G}}}}^{{{\rm{i}}}}}^{{T}}\in {{\mathbb{R}}}^{B\times {C}_{{{\rm{i}}}}},{P}^{{{\rm{v}}}}={\hat{{{\bf{I}}}}}^{{{\rm{v}}}}{{{{\rm{G}}}}^{{{\rm{v}}}}}^{{T}}\in {{\mathbb{R}}}^{B\times {C}_{{{\rm{v}}}}},\quad \\ {P}^{{{\rm{t}}}}={\hat{{{\bf{I}}}}}^{{{\rm{t}}}}{{{{\rm{G}}}}^{{{\rm{t}}}}}^{{T}}\in {{\mathbb{R}}}^{B\times {C}_{{{\rm{t}}}}},{P}^{{{\rm{ivt}}}}={\hat{{{\bf{I}}}}}^{{{\rm{ivt}}}}{{{{\rm{G}}}}^{{{\rm{ivt}}}}}^{{T}}\in {{\mathbb{R}}}^{B\times {C}_{{{\rm{ivt}}}}}.\quad \end{array}\right.$$

We use binary cross-entropy loss^56^, e.g, ${{{\mathcal{L}}}}_{{{\rm{i}}}}$ following:

17 $${{{\mathcal{L}}}}_{{{\rm{i}}}}=\sum\limits_{c=1}^{{C}_{{{\rm{i}}}}}\frac{-1}{{C}_{{{\rm{i}}}}}\left({y}_{c}^{{{\rm{i}}}}\log \left(\sigma \left({P}_{c}^{{{\rm{i}}}}\right)\right)+\left(1-{y}_{c}^{{{\rm{i}}}}\right)\log \left(1-\sigma \left({P}_{c}^{{{\rm{i}}}}\right)\right)\right),$$

where *C*_i_ refers to total number of instrument classes, ${y}_{c}^{{{\rm{i}}}}$ and ${P}_{c}^{{{\rm{i}}}}$ are the ground truth label and the prediction for instrument, *σ* the sigmoid activation function^57^. Similarly, we can obtain ${{{\mathcal{L}}}}_{{{\rm{v}}}}$, ${{{\mathcal{L}}}}_{{{\rm{t}}}}$. For ${{{\mathcal{L}}}}_{{{\rm{ivt}}}}$, as illustrated in Fig. 2 and Supplementary Fig. 2, the proposed I^2^TM in this paper calibrates the predictions of the triplet branch jointly after obtaining predictions for the instrument, verb, and target branches. In contrast to Forest GCN^19^, we conduct a more refined calibration, as follows:

18 $$\left\{\begin{array}{l}{{s}_{c}}^{{{\rm{ivt}}}}=\sigma \left({P}_{c}^{{{\rm{i}}}}\right)\times \sigma \left({P}_{c}^{{{\rm{v}}}}\right)\times \sigma \left({P}_{c}^{{{\rm{t}}}}\right)\times \sigma \left({P}_{c}^{{{\rm{ivt}}}}\right),\quad \\ {{{\mathcal{L}}}}_{{{\rm{ivt}}}}=\sum\limits_{c=1}^{{C}_{{{\rm{ivt}}}}}\frac{-1}{{C}_{{{\rm{ivt}}}}}\left({y}_{c}^{{{\rm{ivt}}}}\log \left({{s}_{c}}^{{{\rm{ivt}}}}\right)+\left(1-{y}_{c}^{{{\rm{ivt}}}}\right)\log \left(1-{{s}_{c}}^{{{\rm{ivt}}}}\right)\right),\quad \end{array}\right.$$

where ${{s}_{c}}^{{{\rm{ivt}}}}$ represents the final predicted score for a given triplet class. Finally, we create the total loss: ${{{\mathcal{L}}}}_{{{\rm{total}}}}={{{\mathcal{L}}}}_{{{\rm{i}}}}+{{{\mathcal{L}}}}_{{{\rm{v}}}}+{{{\mathcal{L}}}}_{{{\rm{t}}}}+{{{\mathcal{L}}}}_{{{\rm{ivt}}}}$.

### Dataset

In our framework, the visual branch is pre-trained on the ImageNet-1K dataset^58^, while the textual branch is pre-trained on the CLIP dataset^53^. Here, the ImageNet-1K dataset consists of over one million images spanning 1000 different classes, which is a widely-used dataset for visual recognition tasks^59,60^. Pre-training on ImageNet-1K allows the model to learn rich and diverse visual features, aiding in the capture of intricate patterns, textures, and object structures. This leads to improved generalization, faster convergence during fine-tuning, and enhanced performance on various visual recognition benchmarks. The CLIP dataset^53^ comprises 400 million image-text pairs collected from the internet, is designed to align visual and textual representations in a shared embedding space. Pre-training on the CLIP dataset^53^ enables the model to map textual descriptions to visual concepts effectively, which is crucial for tasks involving both modalities^61,62^. This results in improved cross-modal understanding, robust multi-modal representations, and better transfer learning capabilities, allowing the model to adapt to a variety of downstream tasks with minimal additional training. Overall, the dual pre-training approach on ImageNet1K and the CLIP dataset enriches our model with comprehensive and well-aligned representations, significantly enhancing its generalization, convergence speed, and overall performance on multi-modal tasks.

In this study, we use two challenging intraoperative video datasets of laparoscopic cholecystectomy procedures for training and testing. The CholecT50 dataset^26^ includes 50 laparoscopic cholecystectomy video sequences recorded at a frequency of 1 fps, generating a total of 100.9K frames and 161K triplet instance labels. Each frame is annotated with 100 binary action triplets, consisting of 6 instruments, 10 verbs, and 15 targets. To evaluate the effectiveness of the proposed framework, we use three different split (i.e., 5-fold cross-validation split, CholecTriplet Challenge split, and RDV split) for model evaluation^26^. In the cross-validation split, we use the more widely utilized CholecT50 subset CholecT45^26^, which is divided into three subsets: a training set consisting of 31 videos, a validation set consisting of 5 videos, and a testing set consisting of 9 videos. The Cholec80 dataset^63^ includes 80 videos with a resolution of 1920 × 1080 or 854 × 480 pixels, recorded at a speed of 25 frames per second (fps). Following previous works, we downsample the dataset to 1 fps. Each frame in the video is manually assigned to one of the seven surgical stages.

### Metrics

We mainly follow previous works in using Average Precision (AP) to measure the accuracy of estimates^26^, which is defined as the area under the precision-recall curve:

19 $$AP=\int_{0}^{1}p(r)dr$$

AP summarizes a p-r curve as the weighted mean of precisions achieved at each threshold, with the increase in recall from the previous threshold used as the weight. The AP metric employed in this study comprises three key aspects: triplet average precision *AP*_*IVT*_, association average precision (*AP*_*IV*_ and *AP*_*IT*_), and component average precision (*AP*_*I*_, *AP*_*V*_, and *AP*_*T*_). The main metric in this study is *AP*_*IVT*_, which directly evaluates the recognition of the complete triplets. *AP*_*IV*_ and *AP*_*IT*_ reflect the performance of instrument-target interaction recognition.

### Model training and testing

All experiments are conducted using PyTorch on a single NVIDIA RTX 4090 GPU. We resize all video frames to a uniform dimension of 256 × 448 following previous works^17–21^. The input length of the video clip is set to 6, and the input video frame is enhanced by light data augmentations, such as flipping, rotation, brightness, and saturation perturbations. For Intra-TSE and Inter-TSE, we utilize ResNet50 for extracting the visual features and generating visual feature prototypes. In Intra-TSE, we extract the output of the last block of ResNet50 as the feature output. In CSTM, the hidden dimension is set as 512, and T-trans follows the architecture of ActionCLIP^23^ with a 6-layer Temporary transformer. For Inter-TSE, we employ a pre-trained CLIP model as the text encoder to extract 512-dimensional text features. During the training process, we set the batch size as 16, and utilize an SGD optimizer with an initial learning rate of 1e-2, which decreases with a cosine learning rate decay schedule. To ensure stable training, we utilize the warmup strategy for the training.

## Supplementary information

Supplementary Materials

## Data Availability

The dataset used in this study can be queried and downloaded from the following public link:https://camma.unistra.fr/datasets/,https://github.com/CAMMA-public/cholect50,https://github.com/CAMMA-public/cholect45.The codes will be available at (https://github.com/halamadrid-lpp/Surgical-video-workflow-analysis-I2TM).

## Code availability

The codes will be available at (https://github.com/halamadrid-lpp/Surgical-video-workflow-analysis-I2TM).
