# Supplementary material for: Surgical video workflow analysis via visual-language learning
Source: Npj Health Syst. 2025 Jan 25;2:5. doi: 10.1038/s44401-024-00010-3 (PMC13354236; doi:10.1038/s44401-024-00010-3)
Supplement: Supplementary file 1 — Supplementary Materials [file 44401_2024_10_MOESM1_ESM.pdf]

Supplementary Materials for

Surgical Video Workflow Analysis via Visual-Language Learning

CONTENTS

The following items are included in the supplementary material:

- Supplementary Figure 1 to Supplementary Figure 3
- Supplementary Table 1 to Supplementary Table 8

SUPPLEMENTARY FIGURES

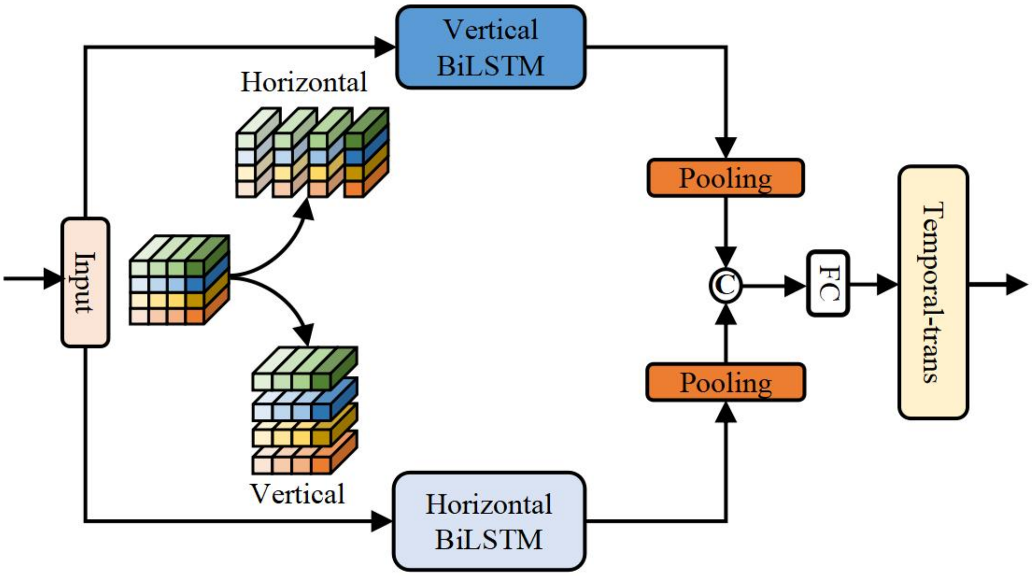

Supplementary Figure 1. Architecture of Coordinate Spatio-Temporal Module (CSTM). To better explore potential representations of visual semantics, we utilize a bidirectional LSTM (BiLSTM) to model the sequence of features obtained along the horizontal and vertical directions. Then, considering the temporal relationship characteristics of surgical videos, we combine the temporal transformer to propose CSTM.

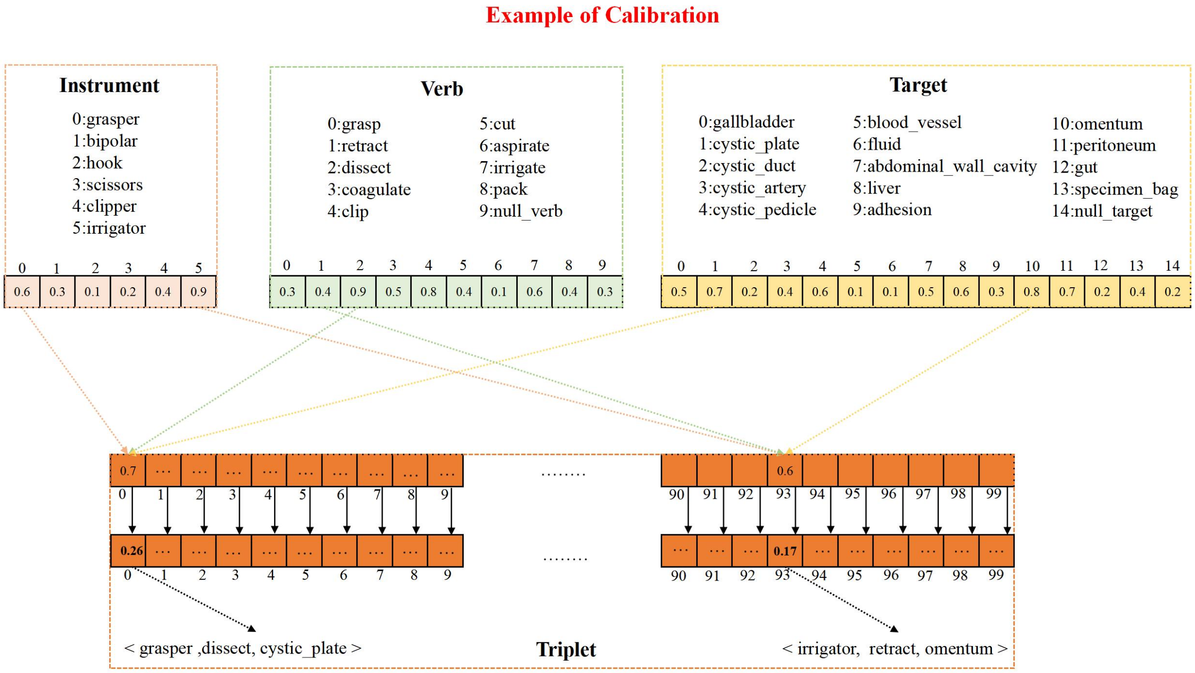

Supplementary Figure 2. Example of Triplet Calibration. Triplet calibration is performed using the following formula: $s_c^{\text{ivt}} = \sigma\left(P_c^{\text{i}}\right) \times \sigma\left(P_c^{\text{v}}\right) \times \sigma\left(P_c^{\text{t}}\right) \times \sigma\left(P_c^{\text{ivt}}\right)$, where P_c^{i}, P_c^{v}, P_c^{t} denote the prediction for instrument, verb, and target, respectively. σ denotes the sigmoid activation function. s_c^{ivt} denotes the final predicted score for a given triplet class. Taking the triplet of < *grasper*, *dissect*, *cystic_plate* > as an example, their predicted probabilities are jointly calibrated with each others.

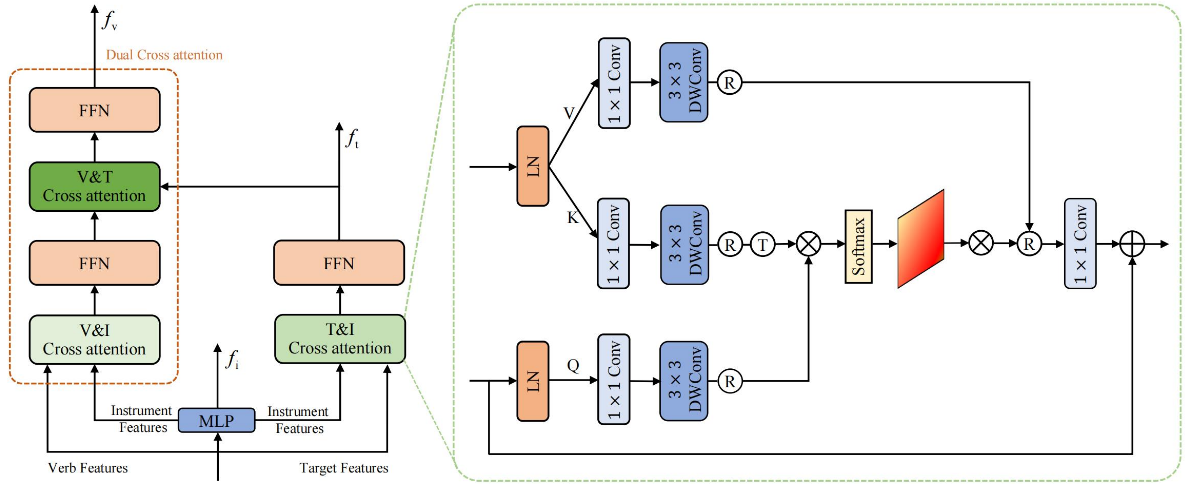

Supplementary Figure 3. Architecture of Triplet Interaction (TI). It obtains preliminary instrument features, target features, and verb features through intra-triplet interaction.

SUPPLEMENTARY TABLES

Supplementary Table 1. Triplet Recognition Average Precision (AP)(%) on CholecT45 dataset using 5-fold cross-validation split.

Method	Visual Backbone	AP_I	AP_V	AP_T	AP_{IV}	AP_{IT}	AP_{IVT}
Tripnet	Resnet18	89.9±1.0	59.9±0.9	37.4±1.5	31.8±4.1	27.1±2.8	24.4±4.7
Attention Tripnet	Resnet18	89.1±2.1	61.2±0.6	40.3±1.2	33.0±2.9	29.4±1.2	27.2±2.7
RDV	Resnet18	89.3±2.1	62.0±1.3	40.0±1.4	34.0±3.3	30.8±2.1	29.4±2.8
RiT	Resnet18	88.6±2.6	64.0±2.5	43.4±1.4	38.3±3.5	36.9±1.0	29.7±2.6
**I²TM**	Resnet18	89.9±1.6	66.5±2.1	43.8±1.6	41.2±3.3	40.0±1.8	33.8±1.9
ConceptNet	Resnet50	84.1±2.9	58.7±1.3	42.4±2.3	38.6±2.0	29.8±1.7	30.1±2.8
ConceptNet	VIT	88.3±1.4	65.1±3.9	43.8±3.1	33.7±3.2	33.4±3.2	30.6±1.9
TD	I3D-Resnet50	91.2±1.9	65.3±2.8	43.7±1.6	–	–	33.8±2.5
**I²TM**	Resnet50	**91. 6±1. 5**	**68.8±2.1**	**45.9±2.7**	**45.2±5.0**	**43.7±2.6**	**37.5±3.9**

Supplementary Table 2. Triplet Recognition AP(%) on CholecT45 dataset using CholecTriplet Challenge split.

Method	AP_I	AP_V	AP_T	AP_{IVT}	Mean
RDV-Det	78.2	46.5	35.9	29.0	47.4
ResNet-CAM-YOLOv5	80.3	50.3	38.4	29.0	49.5
SurgNet	78.8	55.7	40.3	34.4	52.3
MTTT	**84.0**	49.5	40.3	34.5	52.1
Distilled-Swin-YOLO	83.8	52.0	**45.9**	35.0	54.2
**I²TM**	83.1	**56.2**	43.4	**38.1**	**55.2**

Supplementary Table 3. Triplet Recognition AP(%) on CholecT45 dataset using RDV split.

Method	AP_I	AP_V	AP_T	AP_{IV}	AP_{IT}	AP_{IVT}
Naive CNN	57.7	39.2	28.3	21.7	18.0	13.6
TCN	48.9	29.4	21.4	17.7	15.5	12.4
MLT	84.5	28.4	28.2	26.6	21.2	17.6
Tripnet	92.1	54.5	33.2	29.7	26.4	20.0
Attention Tripnet	87.9	59.7	40.6	34.2	29.0	23.2
RDV	92.0	60.7	38.3	39.4	36.9	29.9
Forest GCN	93.1	60.1	40.2	36.2	37.5	36.7
CoT	**94.1**	62.5	41.9	41.7	39.5	38.2
**I²TM**	92.3	**63.6**	**43.1**	**44.1**	**41.3**	**40.2**

Supplementary Table 4. Top K accuracy of the triplet predictions using CholecTriplet Challenge split.

Method	TOP 5	TOP 10	TOP 15	TOP 20	TOP {5:20}
SJTU-IMR	66.50	81.88	84.19	84.89	79.37
SIAT-CAMI	66.58	81.93	88.59	91.84	82.24
HFUT-NUS	65.71	84.18	88.68	90.43	82.25
Digital-Surgery	65.97	81.56	88.78	92.92	82.31
Ceaiik	66.02	81.34	89.74	93.40	82.63
Trequartista	68.50	82.40	88.24	92.29	82.86
Attention-Tripnet	66.86	82.49	91.85	93.25	83.61
HFUT-MedIA	65.05	85.35	91.75	93.59	83.94
Tripnet	67.89	83.99	90.76	93.65	84.07
RDV	69.35	84.38	89.93	93.24	84.23
**I²TM**	**77.29**	**89.78**	**93.17**	**95.59**	**88.96**

Supplementary Table 5. Additional ablation study for Intra-TSE design.

Method	Visual Backbone	AP_I	AP_V	AP_T	AP_{IV}	AP_{IT}	AP_{IVT}
A0	Resnet18	88.6±1.4	64.8±2.3	39.9±3.4	38.7±3.2	38.6±2.0	33.0±1.5
A1	Resnet18	89.6±2.4	64.2±2.1	41.0±2.7	39.9±1.9	39.2±0.5	32.4±1.5
**I²TM(Ours)**	Resnet18	89.9±1.6	66.5±2.1	43.8±1.6	41.2±3.3	40.0±1.8	33.8±1.9

Where A0 and A1 respectively denote the integration of V&I and V&T Cross Attention are in the same branches, but go through V&T first and then V&I and V&T and T&I are in the same branches.

Supplementary Table 6. Additional ablation study for CSTM design.

Method	AP_I	AP_V	AP_T	AP_{IV}	AP_{IT}	AP_{IVT}
B0	83.9±1.5	60.2±1.0	38.8±2.7	35.6±2.2	33.0±1.4	26.0±2.0
B1	86.2±2.7	62.7±0.5	39.3±1.2	36.7±3.4	33.0±2.6	26.1±1.5
B2	86.8±2.3	62.7±2.9	40.1±2.4	38.6±2.8	35.1±1.2	28.1±1.8
**B3**	**87.4±2.6**	**63.3±1.9**	**41.3±3.1**	**40.5±4.6**	**37.3±1.9**	**30.9±3.0**

Where B0 denotes ResNet18 as the base model, B1 denotes (B0+Triplet Interaction), B2 denotes (B1+Temporal-Transformer), and B3 denotes (B1+BiLSTM+Temporal-Transformer).

Supplementary Table 7. Model inference speed data.

Method	Visual Backbone	Memory(GB)	Params(M)	Latency(ms)	FPS
**I²TM**	Resnet18	11.58	270.41	129.16	7.74
**I²TM**	Resnet50	18.09	284.27	135.55	7.38

The data in Table S7. is obtained through inference calculations using a single NVIDIA 4090 GPU. **Memory (GB)** shows the amount of GPU memory used by the model during inference, measured in gigabytes (GB). **Params (M)** indicates the number of parameters in the model, measured in millions (M). **Latency (ms)** refers to the time taken by the model to complete a single inference, measured in milliseconds (ms). **FPS** indicates the number of inferences processed per second, *i.e.*, the number of frames the model can handle per second.

Supplementary Table 8. Per-class triplet recognition AP (%) on 5-fold cross-validation split.

**Classes**	Tripnet	Attention Tripnet	RDV	**I²TM**
grasper,dissect,cystic-plate	01.5±0.3	01.5±0.4	02.2±1.2	03.1±1.9
grasper,dissect,gallbladder	09.5±9.5	04.6±5.6	05.0±4.3	10.0+9.9
grasper,dissect,omentum	01.3±1.2	05.1±5.4	03.4±4.0	00.9±0.2
grasper,grasp,cystic-artery	01.5±0.3	01.3±0.3	02.0±0.6	13.9±9.9
grasper,grasp,cystic-duct	07.9±4.9	12.8±6.5	20.8±9.9	14.0±3.3
grasper,grasp,cystic-pedicle	05.2±1.9	20.1±0.1	02.8±0.8	40.9+9.9
grasper,grasp,cystic-plate	20.3±9.9	21.6±9.9	23.8±9.9	26.5±9.9
grasper,grasp,gallbladder	23.8±9.9	22.2±9.9	30.5±9.9	34.6±9.9
grasper,grasp,gt	00.4±0.1	00.9±0.1	00.3+0.1	00.8±0.1
grasper,grasp,liver	02.5±2.0	02.3±3.1	16.9±9.9	06.9±5.2
grasper,grasp,omentum	04.5±5.8	06.1±4.2	26.7±9.9	25.6±9.9
grasper,grasp,peritoneum	09.2±9.9	04.2±4.7	03.0±2.4	04.2+2.4
grasper,grasp,specimen-bag	85.3±2.3	85.7±1.9	84.5±1.1	89.2±1.8
grasper,pack,gallbladder	30.9±9.9	33.9±9.9	35.2±9.3	52.6±9.9
grasper,retract,cystic-duct	26.9±0.1	00.0±0.0	45.0±0.1	52.2+0.1
grasper,retract,cystic-pedicle	00.8±0.1	02.1±0.1	01.4+0.1	18.8±0.1
grasper,retract,cystic-plate	16.0±9.9	15.9±1.1	17.8±1.3	21.4+8.0
grasper,retract,gallbladder	83.4±7.6	86.5±4.4	83.9±9.6	84.1±8.8
grasper,retract,gut	08.5±5.2	10.5±6.0	10.8±5.1	16.0±5.7
grasper,retract,liver	69.7±6.7	72.1±6.8	72.0±2.6	73.1±6.5
grasper,retract,omentum	44.9±9.9	43.0±9.9	45.5±9.9	48.9+9.9
grasper,retract,peritoneum	17.7±9.9	31.3±9.9	43.5±9.9	56.8±9.9
bipolar,coagulate,abdominal-wall-cavily	41.2±9.9	40.0±9.9	35.6±9.9	56.2±9.9
bipolar,coagulate,blood-vessel	05.5±4.1	12.2+9.1	24.0±9.9	44.4±9.9
bipolar,coagulate,cystic-artery	21.3±1.5	03.9±0.1	15.4±4.9	49.5±9.9
bipolar,coagulate,cystic-duct	02.6±0.1	03.8±0.1	07.7±0.1	16.5±0.1
bipolar,coagulate,cystic-pedicle	27.6±9.9	36.9±9.9	45.5±9.9	55.2±9.9
bipolar,coagulate,cystic-plate	29.3±9.9	25.6±9.9	40.5±9.9	48.7±9.9
bipolar,coagulate,gallbladder	36.6±9.9	52.4±9.9	43.7±9.9	48.7±4.6
bipolar,coagulate,liver	77.6±7.4	79.7±5.9	78.2±6.8	86.0±8.2
bipolar,coagulate,omentum	33.3±9.9	42.8±9.9	37.0±9.9	57.6±9.9
bipolar,coagulate,peritoneum	08.3±0.1	00.0±0.0	22.5±0.1	42.4±0.1
bipolar,dissect,adhesion	07.0±0.1	00.0±0.0	05.1±0.1	30.5±0.1
bipolar,dissect,cystic-artery	07.0±5.1	29.8±9.9	22.3±9.9	28.3±9.9
bipolar,dissect,cystic-duct	25.9±9.9	25.9±3.5	08.9±4.5	14.4±8.5
bipolar.dissect,cystic-plate	04.5±1.9	08.7±0.1	03.5±1.3	10.3±0.4
bipolar,dissect,gallbladder	23.0±9.9	39.3±9.9	20.5±6.3	56.3±9.9
bipolar,dissect,omentum	11.2±0.1	00.0±0.0	26.0±0.1	46.1±0.1
bipolar,grasp,cystic-plate	00.8±0.1	00.3±0.1	00.5±0.1	00.3±0.1
bipolar,grasp,liver	03.4±0.1	95.5±0.1	15.2+0.1	05.2±0.1
bipolar,grasp,specimen-bag	23.3±9.9	25.6±9.9	26.7±9.9	52.7±9.9
bipolar,retract,cystic-duct	00.2±0.1	00.2±0.1	01.7±0.1	05.7±0.1
bipolar,retract,cystic-pedicle	00.8±0.1	37.6±0.1	38.2+0.1	53.6±0.1
bipolar,retract,gallbladder	01.0±0.2	01.9±1.5	01.7±0.7	04.9±1.4
bipolar,retract,liver	15.7±9.9	11.0±5.4	13.3+6.5	23.1±9.9
bipolar,retract,omentum	05.6±4.6	14.8±6.7	17.1±9.4	25.7±9.2
hook,coagulate,blood-vessel	01.2±1.3	00.5±0.1	01.7±1.4	01.6±0.6
hook,coagulate,cystic-artery	00.5±0.1	00.6±0.1	01.3+0.1	05.9±0.1
hook,coagulate,cystic-duct	02.6±3.7	00.5±0.5	02.8±1.8	18.9±9.9

hook,coagulate,cystic-pedicle	00.9±0.4	00.5±0.2	05.6±7.5	00.8±0.3
hook,coagulate,cystic-plate	00.5±0.1	00.0±0.0	00.3±0.1	03.5±0.1
hook,coagulate,gallbladder	06.1±8.2	03.2±1.7	05.6±6.8	19.7±9.9
hook,coagulate,liver	01.9±1.2	02.5±2.1	07.5±4.8	23.7±6.0
hook,coagulate,omentum	07.6±7.6	10.3±6.1	06.3±7.4	15.5±9.9
hook,cut,blood-vessel	00.0±0.0	00.0±0.0	00.0±0.0	00.0±0.0
hook,cut,peritoneum	00.0+0.0	00.0±0.0	00.0±0.0	00.0±0.0
hook,dissect,blood-vessel	00.7±0.1	01.2±0.1	00.9±0.1	10.5±0.1
hook,dissect,cystic-artery	20.4±4.9	19.7±6.6	20.7±4.3	36.7±9.9
hook,dissect,cystic-duct	37.4±4.4	38.7±3.6	39.1±3.1	16.5±2.0
hook,dissect,cystic-plate	14.4+6.5	11.5±5.1	18.3±9.9	25.8±7.5
hook,dissect,gallbladder	78.7±2.6	78.3±3.6	78.3±2.2	74.0±5.6
hook,dissect,omentum	62.5±9.9	65.1±7.0	63.9±8.6	72.5±8.0
hook,dissect,peritoneum	15.1±2.8	11.9±8.5	27.3±3.8	23.9±9.9
hook,retract,gallbladder	14.8±9.9	17.0±5.8	23.8±9.9	28.2±9.9
hook,retract,liver	05.0±6.5	12.1±3.4	19.2+9.9	05.9±5.3
scissors,coagulate,omentum	00.8+0.1	04.0±0.1	01.1±0.1	22.7±0.1
scissors,cut,adhesion	07.9±0.1	12.4±0.1	10.4+0.1	27.1+0.1
scissors,cut,blood-vessel	19.1±9.9	36.7±9.9	33.4±9.9	57.1±9.9
scissors,cut,cystic-artery	50.6±9.9	62.1±4.8	57.3±5.6	65.9±6.9
scissors,cut,cystic-duct	51.8±9.9	56.3±5.6	59.0±7.3	66.7±1.4
scissors,cut,cystic-plate	01.5±1.6	16.0±2.5	22.9±6.1	48.4±9.9
scissors,cut,liver	02.1±0.1	14.9±0.1	08.8±0.1	17.7±0.1
scissors,cut,omentum	01.9+0.1	00.0±0.0	07.9±0.1	79.6±0.1
scissors,cut,peritoneum	02.7±0.1	07.4±0.1	42.9±0.1	89.7±0.1
scissors,dissect,cystic-plate	00.4+0.1	00.4±0.1	02.0±0.1	41.6±0.1
scissors,dissect,gallbladder	02.3±0.1	00.0±0.0	03.7±0.1	67.1±0.1
scissors,dissect,omentum	04.5±0.1	15.8±0.1	06.6±0.1	80.2±0.1
clipper,clip,blood-vessel	13.6±5.8	15.5±9.9	17.4±9.9	36.0±9.9
clipper,clip,cystic-artery	58.7±4.1	61.2±9.9	66.5±4.0	68.0±9.2
clipper,clip,cystic-duct	65.2±9.3	70.0±5.7	70.7±6.1	76.2±9.9
clipper,clip,cystic-pedicle	03.5±0.1	05.2±0.1	26.8±0.1	24.8±0.1
clipper,clip,cystic-plate	02.4+0.9	12.0±9.3	16.3±9.1	47.6±9.9
irrigator,aspirate,fluid	58.9±9.9	57.3±3.0	57.4+9.9	69.0±5.1
irrigator,dissect,cystic-duct	04.7±0.1	00.0±0.0	18.1+0.1	06.8+0.1
irrigator,dissect,cystic-pedicle	18.8±0.5	39.5±9.9	60.6±9.9	66.9±9.9
irrigator,dissect,cystic-plate	01.2+0.1	02.5±0.1	02.0±0.1	35.9±0.1
irrigator,dissect,gallbladder	02.3±2.0	11.1±7.5	19.6±9.9	06.6±1.8
irrigator,dissect,omentum	03.4±3.4	13.5±9.9	08.8±4.1	21.2±9.9
irrigator,irrigate,abdominal-wall-cavity	23.0±9.9	17.8±9.9	28.2±5.8	31.6±9.9
irrigator,irrigate,cystic-pedicle	92.4±2.6	96.1±6.0	92.8±2.0	03.7±1.7
irrigator,irrigate,liver	13.3±9.9	26.9±9.9	18.0±8.0	28.5±9.9
irrigator,retract,gallbladder	19.1±9.9	21.9±9.9	48.5±9.9	19.9±9.9
irrigator,retract,liver	16.7±3.2	24.7±9.9	27.5±6.0	25.4+6.1
irrigator,retract,omentum	05.1±6.7	02.8±2.7	11.0±9.9	09.2±4.0
grasper,null-verb,null-target	22.6±4.8	23.0±4.9	24.4±5.6	26.8±5.1
bipolar,null-verb,null-target	13.0±4.0	14.8±7.7	14.0±7.4	19.8±9.9
hook,null-verb,mull-target	15.8±4.6	17.5±2.3	17.0±2.7	23.3±3.0
scissors,null-verb,null-target	06.8±2.5	23.9±9.9	15.1+9.9	20.0+9.9
dipper,null-verb,null-target	25.4±9.9	22.6±9.9	33.0±9.9	32.3±9.9
irrigator,null-verb,null-target	15.2±9.0	14.7±6.6	13.1±3.8	29.0±9.9
Mean	24.4±04.7	27.2±02.7	29.4±02.8	**37.5±3.9**
